# Proteomic Response of Three Marine Ammonia-Oxidizing Archaea to Hydrogen Peroxide and Their Metabolic Interactions with a Heterotrophic Alphaproteobacterium

**DOI:** 10.1128/mSystems.00181-19

**Published:** 2019-06-25

**Authors:** Barbara Bayer, Claus Pelikan, Meriel J. Bittner, Thomas Reinthaler, Martin Könneke, Gerhard J. Herndl, Pierre Offre

**Affiliations:** a Department of Limnology and Bio-Oceanography, Centre of Functional Ecology, University of Vienna, Vienna, Austria; b Division of Microbial Ecology, Centre for Microbiology and Environmental Systems Science, University of Vienna, Vienna, Austria; c Marine Archaea Group, MARUM—Center for Marine Environmental Sciences & Department of Geosciences, University of Bremen, Bremen, Germany; d Department of Marine Microbiology and Biogeochemistry, NIOZ Royal Netherlands Institute for Sea Research and Utrecht University, Den Burg, Texel, The Netherlands; e Vienna Metabolomics Center, University of Vienna, Vienna, Austria; Woods Hole Oceanographic Institution

**Keywords:** *Nitrosopumilus*, ammonia-oxidizing archaea, hydrogen peroxide, metabolic interactions, oxidative stress, proteomics

## Abstract

Ammonia-oxidizing archaea (AOA) play an important role in the nitrogen cycle and account for a considerable fraction of the prokaryotic plankton in the ocean. Most AOA lack the hydrogen peroxide (H_2_O_2_)-detoxifying enzyme catalase, and some AOA have been shown to grow poorly under conditions of exposure to H_2_O_2_. However, differences in the degrees of H_2_O_2_ sensitivity of different AOA strains, the physiological status of AOA cells exposed to H_2_O_2_, and their molecular response to H_2_O_2_ remain poorly characterized. Further, AOA might rely on heterotrophic bacteria to detoxify H_2_O_2_, and yet the extent and variety of costs and benefits involved in these interactions remain unclear. Here, we used a proteomics approach to compare the protein profiles of three *Nitrosopumilus* strains grown in the presence and absence of catalase and in coculture with the heterotrophic alphaproteobacterium Oceanicaulis alexandrii. We observed that most proteins detected at a higher relative abundance in H_2_O_2_-exposed *Nitrosopumilus* cells had no known function in oxidative stress defense. Instead, these proteins were putatively involved in the remodeling of the extracellular matrix, which we hypothesize to be a strategy limiting the influx of H_2_O_2_ into the cells. Using RNA-stable isotope probing, we confirmed that O. alexandrii cells growing in coculture with the *Nitrosopumilus* strains assimilated *Nitrosopumilus*-derived organic carbon, suggesting that AOA could recruit H_2_O_2_-detoxifying bacteria through the release of labile organic matter. Our results contribute new insights into the response of AOA to H_2_O_2_ and highlight the potential ecological importance of their interactions with heterotrophic free-living bacteria in marine environments.

**IMPORTANCE** Ammonia-oxidizing archaea (AOA) are the most abundant chemolithoautotrophic microorganisms in the oxygenated water column of the global ocean. Although H_2_O_2_ appears to be a universal by-product of aerobic metabolism, genes encoding the hydrogen peroxide (H_2_O_2_)-detoxifying enzyme catalase are largely absent in genomes of marine AOA. Here, we provide evidence that closely related marine AOA have different degrees of sensitivity to H_2_O_2_, which may contribute to niche differentiation between these organisms. Furthermore, our results suggest that marine AOA rely on H_2_O_2_ detoxification during periods of high metabolic activity and release organic compounds, thereby potentially attracting heterotrophic prokaryotes that provide this missing function. In summary, this report provides insights into the metabolic interactions between AOA and heterotrophic bacteria in marine environments and suggests that AOA play an important role in the biogeochemical carbon cycle by making organic carbon available for heterotrophic microorganisms.

## Abstract (precis)

Ammonia-oxidizing archaea (AOA) are the most abundant chemolithoautotrophic microorganisms in the oxygenated water column of the global ocean. Although H_2_O_2_ appears to be a universal by-product of aerobic metabolism, genes encoding the hydrogen peroxide (H_2_O_2_)-detoxifying enzyme catalase are largely absent in genomes of marine AOA. Here, we provide evidence that closely related marine AOA have different degrees of sensitivity to H_2_O_2_, which may contribute to niche differentiation between these organisms. Furthermore, our results suggest that marine AOA rely on H_2_O_2_ detoxification during periods of high metabolic activity and release organic compounds, thereby potentially attracting heterotrophic prokaryotes that provide this missing function. In summary, this report provides insights into the metabolic interactions between AOA and heterotrophic bacteria in marine environments and suggests that AOA play an important role in the biogeochemical carbon cycle by making organic carbon available for heterotrophic microorganisms.

## INTRODUCTION

Ammonia-oxidizing archaea (AOA) are a major component of marine microbial communities and represent the dominant ammonia oxidizers in the ocean, carrying out the first and rate-limiting step of nitrification (1–3). AOA are members of the phylum Thaumarchaeota (4, 5) and are particularly abundant in the mesopelagic zone of the open ocean (6) and in oxygen minimum zones (7).

Although more than a dozen strains of autotrophic AOA have been enriched from marine waters and sediments (8–14), their isolation and maintenance on a mineral medium have repeatedly proven difficult. To some extent, this difficulty may be attributed to the dependence of some AOA on the presence of alpha-ketoacids or, alternatively, that of cocultivated heterotrophic bacteria to achieve exponential growth in batch cultures (9, 15, 16). Recently, this dependency has been linked to their sensitivity to hydrogen peroxide (H_2_O_2_), which is detoxified by alpha-ketoacids and/or cocultivated heterotrophs (14). The sensitivity of AOA to H_2_O_2_ is somewhat surprising as H_2_O_2_ appears to be a universal by-product of aerobic metabolism and the vast majority of aerobic organisms encode H_2_O_2_-scavenging enzymes, including catalases and peroxidases (17, 18). The H_2_O_2_ sensitivity reported for some AOA strains isolated from marine environments correlates with the lack of genes encoding canonical catalase homologs in their genomes (14, 19).

In the ocean, H_2_O_2_ is mainly produced by the photooxidation of chromophoric dissolved organic matter (20) but is also introduced via precipitation (21) and metabolic processes (22, 23). The lack of a recognizable form of H_2_O_2_ detoxification machinery in AOA was hypothesized to result from their limited exposure to this oxidant under oligotrophic conditions (14). However, in marine surface waters, AOA might be chronically exposed to H_2_O_2_, where its concentrations can reach up to 500 nM (24). While the ammonia oxidization activity of the marine archaeon *Nitrosopumilus* strain DDS1 was completely inhibited after production of ∼200 nM H_2_O_2_ (14), Nitrosopumilus maritimus SCM1 showed no decrease in ammonia oxidation after additions of H_2_O_2_ (up to 1 μmol liter^−1^) to the culture medium (25). Differences in H_2_O_2_ sensitivity have also been reported for environmental AOA populations and were suggested previously to be defining features of distinct AOA ecotypes (19). These observations indicate that the degrees of H_2_O_2_ tolerance differ across the vast diversity of AOA species, and yet exploration of the entire spectrum of their phenotypic response to H_2_O_2_ has just started. Furthermore, it is unclear whether the inability to achieve exponential growth in the absence of an external H_2_O_2_ scavenger (i.e., alpha-ketoacids [14–16]) represents a consequence or the absence of a molecular response to H_2_O_2_.

In this study, we compared the growth levels of three strains of marine AOA in the presence and absence of commercial catalase or under conditions of growth in coculture with the heterotrophic alphaproteobacterium Oceanicaulis alexandrii and evaluated the concurrent levels of production and eventual scavenging of H_2_O_2_ in the culture medium. The investigated AOA comprised all marine axenic cultures with closed genomes which are currently available, including the first reported AOA isolate, Nitrosopumilus maritimus SCM1 (26), as well as two isolates from coastal surface waters of the Northern Adriatic Sea, Nitrosopumilus adriaticus NF5 and Nitrosopumilus piranensis D3C (27). O. alexandrii was the most persistent contaminant prior to obtaining axenic cultures of N. adriaticus NF5 and N. piranensis D3C (9). Furthermore, the molecular response of the investigated strains to H_2_O_2_ was assessed by comparing the proteomes of cells growing in the presence or in the absence of catalase and/or the alphaproteobacterium O. alexandrii. Additionally, metabolic interactions of the three *Nitrosopumilus* strains with O. alexandrii were explored using stable isotope probing and comparative proteome analysis.

Collectively, the results of this study provide insights into the molecular and physiological responses of AOA to H_2_O_2_ and highlight the potential ecological importance of their interactions with heterotrophic free-living bacteria in marine environments.

## RESULTS AND DISCUSSION

### The effect of H_2_O_2_ on the growth and ammonia oxidation activity of three *Nitrosopumilus* strains.

**FIG 1:**
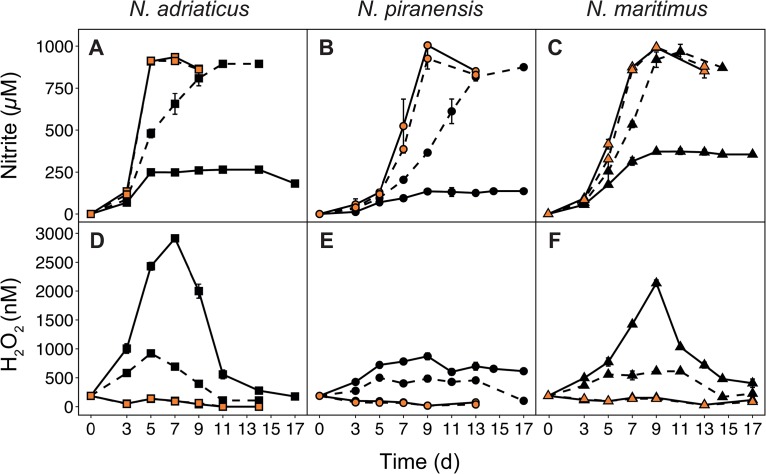
Nitrite production (A, B, and C) and hydrogen peroxide (H_2_O_2_) concentrations (D, E, and F) of three *Nitrosopumilus* strains grown in pure culture (solid line) or in coculture with O. alexandrii (dashed line). Orange shapes indicate the addition of catalase. Error bars represent standard deviations of measurements from triplicate cultures. d, days.

Ammonia oxidation activity and growth of three *Nitrosopumilus* strains (N. adriaticus NF5, N. piranensis D3C, and N. maritimus SCM1) were assessed both in the presence and absence of commercial catalase and under conditions of growth in coculture with the heterotrophic alphaproteobacterium Oceanicaulis alexandrii. When catalase was added to the cultures, all three *Nitrosopumilus* strains depleted 1 mM ammonium within 5 to 8 days of incubation (Fig. 1A to C), yielding nearly stoichiometric amounts of nitrite as previously described for these strains (9, 25). In cocultures with O. alexandrii, however, nitrite production was consistently slower than in axenic cultures supplemented with catalase. In cocultures, the complete conversion of ammonium to nitrite took 10 to 15 days, possibly due to the proportionally small O. alexandrii cell population size and consequently low H_2_O_2_-detoxifying capacity of O. alexandrii relative to purified catalase. Adding both catalase and O. alexandrii did not result in an increased rate of nitrite production relative to those observed for axenic AOA cultures grown in the presence of catalase.

In the absence of catalase and O. alexandrii, all three *Nitrosopumilus* strains depleted the supplied ammonium only partially and nitrite concentrations remained constant at 150 to 300 μM after 5 to 9 days of incubation (Fig. 1A to C). The stalled production of nitrite in axenic cultures devoid of catalase correlated with a growth arrest of the cultures. Maximum cell abundances ranged between 4 × 10^6^ and 1 × 10^7^ cells ml^−1^ after 3 to 6 days of incubation, which was on average 5 to 10 times lower than in cultures containing catalase (see Table S1 in the supplemental material). Growth arrest was observed 2 to 3 days earlier than the arrest of nitrite production in all three strains, indicating that the remaining level of ammonia oxidation was insufficient to meet the energy demands of dividing cells during H_2_O_2_ exposure (Fig. 1A to C; see also Table S1).

10.1128/mSystems.00181-19.7 TABLE S1 Cell counts of *Nitrospumilus* and Oceanicaulis alexandrii cultures grown in different treatments (CAT, catalase; coculture; without addition of catalase or O. alexandrii). Cell counts were derived from one of three biological replicates per strain and treatment. Download Table S1, DOCX file, 0.02 MB. Copyright © 2019 Bayer et al. 2019 Bayer et al. This content is distributed under the terms of the Creative Commons Attribution 4.0 International license.

In the medium of cultures lacking both catalase and O. alexandrii, H_2_O_2_ concentrations reached 800 nM to 3 μM after 7 to 9 days of incubation (Fig. 1D to F), which was far above the background levels of H_2_O_2_ (∼150 nM) in the culture medium (see Fig. S1A in the supplemental material). Concentrations of H_2_O_2_ remained fairly stable in the abiotic controls (Fig. S1A), indicating that the investigated *Nitrosopumilus* strains produced H_2_O_2_ as a result of their metabolic activity. Although the fluorescence-based assay used in this study (see Materials and Methods) could potentially detect oxidizing agents other than H_2_O_2_, complete loss of the signal after catalase addition suggests that H_2_O_2_ was the primary oxidant measured. Importantly, the presence of the organic buffer HEPES in the culture medium did not appear to represent a dominant source of H_2_O_2_, in contrast to previous reports on phytoplankton cultures (28) (Fig. S1A). Indeed, the HEPES buffer may release H_2_O_2_ only under conditions of exposure to light whereas the three *Nitrosopumilus* strains were grown in the dark. After reaching their peak, H_2_O_2_ concentrations also declined in axenic cultures devoid of catalase (Fig. 1D to F). This decline in the H_2_O_2_ concentration was also observed in the abiotic controls, where H_2_O_2_ was added at ∼3.5 μM (Fig. S1B), suggesting that H_2_O_2_ was not actively scavenged by the investigated strains. In all cultures containing the purified catalase, H_2_O_2_ concentrations never increased above background levels, whereas in cocultures, H_2_O_2_ concentrations sporadically reached levels as high as 900 nM in N. adriaticus-O. alexandrii cocultures (Fig. 1D).

10.1128/mSystems.00181-19.2 FIG S1 (A) Hydrogen peroxide (H_2_O_2_) concentrations of SCM medium controls with or without addition of catalase (5 U ml^−1^) incubated in the dark at 30°C. (B) Abiotic reduction of H_2_O_2_ in SCM medium over time. Error bars represent the range of the mean from measurements of duplicate cultures (sometimes too small to be visible). Download FIG S1, PDF file, 0.2 MB. Copyright © 2019 Bayer et al. 2019 Bayer et al. This content is distributed under the terms of the Creative Commons Attribution 4.0 International license.

Comparing the three strains, complete inhibition occurred at lower H_2_O_2_ concentrations in N. piranensis (∼800 nM; Fig. 1E) than in N. adriaticus and N. maritimus (2.5 μM and 2 μM H_2_O_2_, respectively; Fig. 1D and F). Although N. maritimus produced more nitrite than N. adriaticus prior to inhibition (∼350 μM nitrite versus ∼250 μM, respectively; Fig. 1A and C), N. adriaticus exhibited a higher cell-specific net level of H_2_O_2_ production (Fig. 1; see also Table S1), suggesting that the molecular machinery responsible for H_2_O_2_ production in distinct AOA strains could have different H_2_O_2_ production yields. In contrast to N. piranensis and N. maritimus, which encode two putative superoxide dismutases, N. adriaticus encodes three superoxide dismutases, potentially explaining the higher observed level of H_2_O_2_ production by this strain.

The three strains investigated in this study appeared to tolerate higher concentrations of H_2_O_2_ than *Nitrosopumilus* strain DDS1, which was reported to be completely inhibited at ∼200 nM H_2_O_2_ (14). While strain DDS1 was isolated from a water depth of 200 m, N. adriaticus NF5 and N. piranensis D3C were isolated from coastal surface waters where H_2_O_2_ concentrations are typically ∼10 to 100 times higher than in deeper waters (29, 30). Both strains putatively encode cyclobutane pyrimidine dimer (CPD) photolyase (9), an enzyme activated by UV-A radiation to repair DNA damage (31), suggesting that they might be more tolerant of conditions typically found in surface waters (i.e., higher H_2_O_2_ concentrations) than strains isolated from deeper water layers. N. maritimus was previously observed to be insensitive to additions of H_2_O_2_ and was still able to oxidize 1 mM ammonia when H_2_O_2_ was added to the culture medium at 5 μmol liter^−1^ (25), which contrasts with the complete inhibition of N. maritimus at 2 μM H_2_O_2_ that we report here (Fig. 1F). Considering the different experimental setup and the lack of H_2_O_2_ concentration measurements in the incubations cited above (26), the explanation of the different results that we report here remains currently unclear.

**FIG 2:**
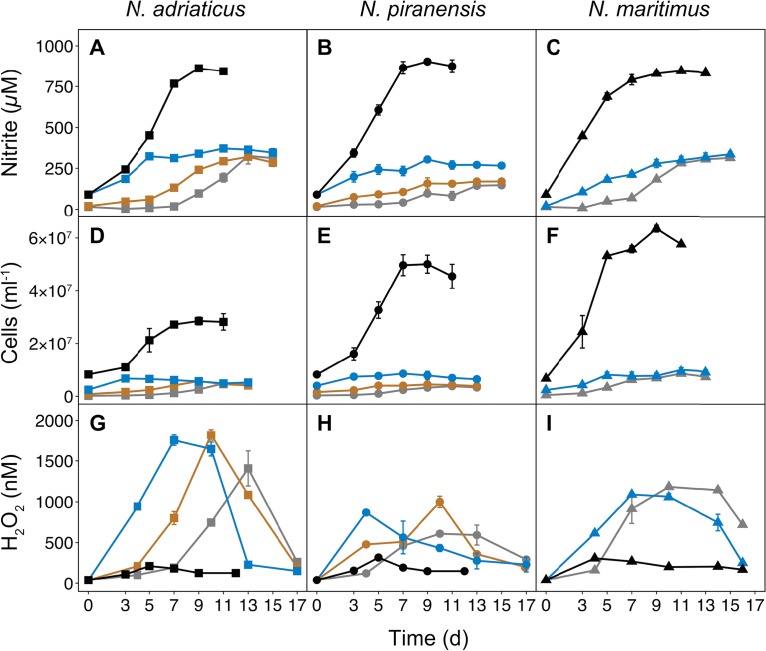
Nitrite concentrations (A, B, and C), cell abundances (D, E, and F), and hydrogen peroxide (H_2_O_2_) concentrations (G, H, and I) of three *Nitrosopumilus* strains grown at various initial cell abundances, which are represented by different colors: ∼2 × 10^5^ ml^−1^ (gray lines), ∼8 × 10^5^ ml^−1^ (brown lines), ∼3 × 10^6^ ml^−1^ (blue lines), ∼7 × 10^6^ ml^−1^ (black lines). Error bars represent the range of the mean from measurements of duplicate cultures.

All three *Nitrosopumilus* strains could overcome H_2_O_2_-induced growth arrest when initial cell abundances were higher than or equal to 7 × 10^6^ ml^−1^, and growth of these cultures was similar to those containing catalase (Fig. 2A to F). While H_2_O_2_ concentrations appeared to increase linearly with nitrite production in cultures with low initial cell abundances (∼2 × 10^5^, ∼8 × 10^5^, and ∼3 × 10^6^ ml^−1^; Fig. 2A, C, and G to I), the detected H_2_O_2_ concentrations were much lower (<300 nM) in cultures with high initial cell abundances (7 × 10^6^ ml^−1^; Fig. 2G to I), indicating either that less H_2_O_2_ was produced or that it was scavenged by an unknown mechanism. Similar cell abundance-dependent H_2_O_2_ sensitivity patterns have been described in axenic *Prochlorococcus* cultures, which grew well in concentrated but not in dilute cultures (32, 33). In the absence of H_2_O_2_ scavengers, growth and ammonia oxidation activity did not show a linear response to the size of the inoculum but rather were induced at a certain cell abundance level in all three *Nitrosopumilus* strains (Fig. 2A to F). Even though knowledge of the exact mechanism of this phenomenon remains elusive, cell abundance-dependent cellular responses are commonly induced by quorum sensing.

While the culture conditions in this study did not reflect the oligotrophic conditions typically found in the ocean, note that all three *Nitrosopumilus* strains also produced large amounts of H_2_O_2_ at environmentally relevant cell abundances of ∼2 × 10^5^ ml^−1^ (Fig. 2D to F). Thus, in their natural environment, marine AOA might potentially rely on H_2_O_2_ detoxification by other microorganisms during periods of high activity and/or in nutrient-rich microniches.

### The proteomic response of three *Nitrosopumilus* strains to H_2_O_2_.

Comparative proteomics was used to investigate the molecular response of the three *Nitrosopumilus* strains to H_2_O_2_ exposure relative to cultures grown in the presence of commercial catalase or in coculture with the heterotrophic bacterium O. alexandrii. Additionally, we distinguished between axenic cultures that were completely inhibited in their growth (H_2_O_2_ inhibited) and axenic cultures that were grown at a high initial cell abundance (7 × 10^6^ ml^−1^) and were able to grow despite the absence of an H_2_O_2_ scavenger (H_2_O_2_ noninhibited).

A total of 1,020 to 1,372 proteins were identified by liquid chromatography-tandem mass spectrometry (LC-MS/MS) and accounted for ∼55% to 68% of the predicted coding DNA sequences in the genomes of N. adriaticus NF5, N. piranensis D3C, and N. maritimus SCM1 (Table S2A). Between the four treatments (with catalase, with O. alexandrii, H_2_O_2_ inhibited, and H_2_O_2_ noninhibited), 1,040, 1,027, and 856 proteins were shared by N. adriaticus, N. piranensis, and N. maritimus, respectively (Table S2A). While the relative abundances of the majority of proteins remained constant between the different treatments, 56 to 109 proteins significantly changed in their relative abundances (increased or decreased; adjusted *P* value, <0.05) in the absence of catalase or O. alexandrii compared to the results seen with cultures grown in the presence of either catalase or O. alexandrii (Table S2B). Of these, 33 proteins were shared among all three strains. Comparing the proteome composition of cultures grown in the presence of catalase to that of cocultures grown with O. alexandrii, 12 to 20 proteins changed in their relative abundances, 5 of which were shared by all three strains (Table S2B). However, these 5 shared proteins also changed in relative abundance when *Nitrosopumilus* cells were exposed to H_2_O_2_, suggesting that they did not represent a specific response to the presence of the heterotrophic bacterium itself. Instead, they might have been induced as a result of the lower capacity of O. alexandrii to detoxify H_2_O_2_ than purified catalase (Fig. 1D to F).

10.1128/mSystems.00181-19.8 TABLE S2 (A) Proteins shared between proteomes of three *Nitrosopumilus* strains grown under conditions of different treatments (with catalase, with O. alexandrii, H_2_O_2_ inhibited, and H_2_O_2_ noninhibited). (B) Significant changes in relative protein abundances of three *Nitrosopumilus* strains based on pairwise comparisons between different treatments. Each treatment included proteins shared by three biological replicates. Download Table S2, DOCX file, 0.01 MB. Copyright © 2019 Bayer et al. 2019 Bayer et al. This content is distributed under the terms of the Creative Commons Attribution 4.0 International license.

Furthermore, despite the reduced sensitivity to H_2_O_2_ in *Nitrosopumilus* cultures with high initial cell abundances (H_2_O_2_ noninhibited) (Fig. 2), we did not detect any changes in the proteome composition shared by all three strains relative to cultures that were completely inhibited by H_2_O_2_ (Table S2B). This suggests that the proteome composition of cells exposed to H_2_O_2_ does not directly indicate whether the cells are active, indicating the existence of further regulatory mechanisms (i.e., noncoding RNAs or posttranslational protein modifications) determining growth. Previous studies have reported that most of the abundant transcripts are relatively invariant across growth phases and environmental conditions in AOA (12, 34).

Several strain-specific changes in the proteome composition were identified across the four treatments in each of the three strains (Fig. S2A to C; see also Text S1 in the supplemental material); however, we focus in the following sections on the proteomic features shared by all three strains.

10.1128/mSystems.00181-19.1 TEXT S1 In-depth descriptions of protocols used for protein extraction, LC-MS/MS analysis, and peptide identification, RNA extraction, quantitative PCR, flow cytometry, and CARD-FISH. Discussion of additional information on extracellular matrix-associated proteins, strain-specific proteomic responses of the individual *Nitrosopumilus* strains to H_2_O_2_, and potential H_2_O_2_ production sites in AOA. Download Text S1, DOCX file, 0.07 MB. Copyright © 2019 Bayer et al. 2019 Bayer et al. This content is distributed under the terms of the Creative Commons Attribution 4.0 International license.

10.1128/mSystems.00181-19.3 FIG S2 Heat map of the proteins that showed a significant change in relative abundance in three *Nitrosopumilus* strains under different culture conditions (with catalase, with O. alexandrii, H_2_O_2_ inhibited, and H_2_O_2_ noninhibited). Columns and rows were clustered based on Euclidean distances corresponding to differences between treatments and relative protein abundances, respectively. (A) Nitrosopumilus adriaticus NF5. (B) Nitrosopumilus piranensis D3C. (C) Nitrosopumilus maritimus SCM1. Download FIG S2, PDF file, 0.8 MB. Copyright © 2019 Bayer et al. 2019 Bayer et al. This content is distributed under the terms of the Creative Commons Attribution 4.0 International license.

**(i) Thaumarchaeal homologs of proteins involved in canonical oxidative stress defense.** Microbial cells use various intracellular scavenging and repair mechanisms to limit and repair damage caused by reactive oxygen species (ROS). In bacteria, the basal scavenging system for O_2_^-^ is superoxide dismutase, whereas H_2_O_2_ is scavenged by catalase and peroxidases (18, 35). Genetic responses to H_2_O_2_ stress in Gram-negative and Gram-positive bacteria are controlled by the transcriptional regulons OxyR and PerR, respectively (36–38), which regulate the expression of catalase, alkyl hydroperoxide reductase (Ahp), and proteins involved in disulfide reduction, heme synthesis, iron scavenging (ferritin and related proteins), and iron import control, as well as proteins involved in divalent cation import (mostly manganese) (see reference 39 and references therein). ROS scavenging systems in archaea, although much less extensively studied, have been shown to be similar to those in bacteria (40, 41).

Although the genomes of all cultured marine AOA do not encode canonical catalase homologs, genes encoding putative catalases have recently been reported from two single-cell genomes obtained from AOA cells sampled from Antarctic surface waters (42). These genes were predicted to be horizontally acquired genes (42). We reanalyzed these single-cell genomes and observed that catalase-encoding genes are part of short contigs that harbor only genes of presumed bacterial origin (due to their similarity to known bacterial homologs), suggesting that they represent contaminating genome fragments that originated from bacterial genomes (see Data Set S2A in the supplemental material). Although there is tangible evidence of the presence of catalase-encoding genes in AOA isolated from terrestrial environments (43, 44) and from a wastewater treatment plant (45), thus far, there is no evidence suggesting that marine AOA encode catalases.

In spite of the notable absence of catalase homologs, we identified homologs of known ROS-scavenging enzymes and, more generally, of proteins involved in canonical pathways of oxidative stress defense in the genomes and proteomes of the three *Nitrosopumilus* strains that we investigated (Data Set S1D). Surprisingly, however, the relative abundances of most proteins assumed to play a role in oxidative stress defense did not change in response to H_2_O_2_ exposure. These also included the five putative Ahp proteins, which raises the issue of whether these putative candidates are functional H_2_O_2_-detoxifying enzymes as previously contested (14) or are generally not regulated on the gene expression level in members of the *Nitrosopumilus* genus. Alternatively, intracellular H_2_O_2_ concentrations might not have been high enough to induce a response.

10.1128/mSystems.00181-19.9 DATA SET S1 (A) Orthologous groups constructed from the genomes of Nitrosopumilus adriaticus NF5, Nitrosopumilus piranensis D3C, and Nitrosopumilus maritimus SCM1. (B) Gene annotations of orthologous groups. (C) KEGG assignments of the H_2_O_2_ responsive proteins shared by all three strains. (D) Relative protein abundances of the proteins related to canonical oxidative stress defense mechanisms in all three *Nitrosopumilus* strains for each treatment. Download Data Set S1, XLSX file, 0.2 MB. Copyright © 2019 Bayer et al. 2019 Bayer et al. This content is distributed under the terms of the Creative Commons Attribution 4.0 International license.

**FIG 3:**
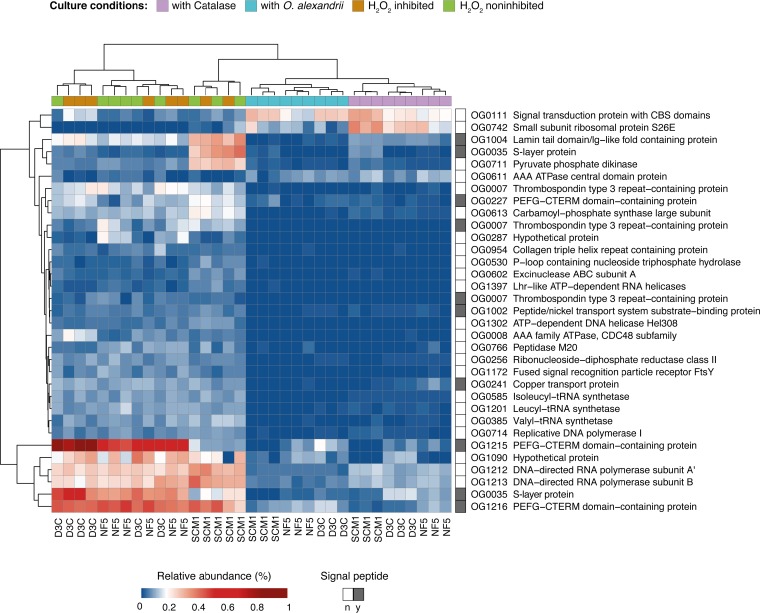
Heat map of proteins that showed a significant change in relative abundance in three *Nitrosopumilus* strains (N. adriaticus NF5, N. piranensis D3C, and N. maritimus SCM1) comparing different culture conditions (with catalase, with O. alexandrii, H_2_O_2_ inhibited, and H_2_O_2_ noninhibited). Columns and rows were clustered based on Euclidean distances corresponding to differences between treatments and relative protein abundances, respectively. The presence of a signal peptide is indicated in dark gray.

Nevertheless, more than one-third (36%) of the proteins that showed a significant response to H_2_O_2_ exposure were assigned to KEGG orthologous groups (OGs) associated with genetic information processing and nucleotide metabolism (Fig. 3; see also Data Set S1C). These included DNA polymerase I and ATP-dependent helicase, which are key enzymes of DNA replication, as well as two subunits of DNA-directed RNA polymerase, which is involved in RNA synthesis (Fig. 3). Additionally, ribonucleotide reductase, which converts ribonucleotides into deoxyribonucleotides, was identified at high relative abundance. While ribonucleotide reductase expression is necessary for DNA replication, it has also been shown to be induced in response to DNA damage and replication blocks (46, 47). Furthermore, proteins putatively involved in the repair of DNA damage, including excinuclease ABC subunit A (UvrA) and DNA helicase Hel308, were identified at high relative abundance during H_2_O_2_ exposure (Fig. 3). The latter is suggested to play a role in the repair and start of replication forks (48), whereas UvrA is part of the nucleotide excision repair mechanism (49). Nucleotide excision repair initiation involves a two-step mechanism in which UvrA initially scans the genome and locates DNA damage prior to recruiting UvrB and UvrC, which are needed for DNA damage verification, excision, and, ultimately, repair (49, 50). However, we did not detect an increase in the relative abundance of UvrBC, suggesting that H_2_O_2_ concentrations in *Nitrosopumilus* cells might not have been high enough to induce extensive DNA damage.

The concentration of H_2_O_2_ inside cells is dependent on the rate of its endogenous formation and influx, balanced against the rate of H_2_O_2_ scavenging and efflux (51). A few studies have suggested previously that extent of passage of H_2_O_2_ through biological membranes is limited (51–53). In Escherichia coli, endogenous H_2_O_2_ is rapidly scavenged by Ahp (and to only a lower extent by catalase) to levels below 20 nM and does not persist long enough to penetrate the membrane, with less than 10% escaping the cell (51, 54). Hence, there is typically no measurable accumulation of H_2_O_2_ in the culture medium (54), in contrast to the range of H_2_O_2_ concentrations of ∼1 to 2.5 μM measured in the *Nitrosopumilus* culture medium in this study. Additionally, putative Ahp proteins have been identified in the proteomes of all three *Nitrosopumilus* strains at similar abundance levels in all treatments as mentioned above. On the basis of these observations, we hypothesize that production of H_2_O_2_ in AOA possibly takes place at the outer side of the membrane (e.g., in the pseudoperiplasmic space).

**(ii) Increase of relative abundances of membrane-associated proteins in response to H_2_O_2_.** Among the proteins that showed a significant change in relative abundance in AOA cultures exposed to H_2_O_2_ (H_2_O_2_ inhibited and noninhibited treatments), ∼30% contained a signal peptide indicating secretion and/or localization on the outer side of the cytoplasmic membrane (Fig. 3). Concomitantly, tRNA synthetases for branched-chain amino acids, essential components of membrane spanning helices and thus of membrane bound/surface proteins (55), were identified at higher relative abundances (Fig. 3). Moreover, the high level of representation of the signal recognition particle (SRP) receptor protein FtsY, which likely mediates the delivery of SRP-nascent chain complexes to the cell membrane (56), suggests an increased rate of transport of proteins to the membrane.

Both putative membrane-bound S-layer proteins were among the 10 to 50 most abundant proteins detected in axenic cultures devoid of catalase (Fig. 3). The putative S-layer proteins were on average 4 to 30 times more abundant in the proteomes of axenic cultures grown in the absence of catalase than in those of cultures grown in the presence of catalase or in coculture with O. alexandrii, potentially indicating an increased renewal or restructuring of the S-layer coat under conditions of H_2_O_2_ exposure. In addition, three PEFG-CTERM domain-containing proteins were detected at 4 to 10 times higher relative abundance in axenic cultures without addition of catalase than cultures grown with catalase or in coculture with O. alexandrii. The PEFG-CTERM motif resembles the PEP-CTERM domain typically present in glycoproteins which are transported and anchored into the plasma membrane, such as S-layer proteins in Haloferax volcanii (57). Furthermore, two thrombospondin type 3-like repeat (TT3R)-containing proteins were on average 15 to 40 times more abundant in cultures grown under conditions of H_2_O_2_ exposure than in cultures containing catalase or O. alexandrii (Fig. 3). Proteins containing TT3R motifs in AOA show sequence similarity to hypothetical proteins of the myxobacterial thrombospondin-like gene cluster, which has been suggested to play a role in the construction of the cell surface matrix (58). However, besides the well-known calcium-binding capacities of TT3R motifs, knowledge of their exact functions in prokaryotes remains elusive (59). Nevertheless, we detected putative structural proteins (OG1004 and OG0954) which share homology with proteins known to interact with thrombospondins in eukaryotes at high relative abundance levels in all three *Nitrosopumilus* strains under conditions of exposure to H_2_O_2_ (Fig. 3) (further discussed in Text S1 in the supplemental material).

The increase in the relative abundance of putative components of the extracellular matrix of AOA is reminiscent of the protective barrier formed by the eukaryote Saccharomyces cerevisiae to limit the influx of H_2_O_2_ into its cells (60). Furthermore, cell aggregation, exopolysaccharide (EPS) production, and, ultimately, biofilm formation represent common physiological responses of bacteria exposed to H_2_O_2_ and may promote survival (61, 62). AOA species, including members of the *Nitrosopumilus* genus, contain the genomic repertoire for exopolysaccharide production and cell surface modifications (63). Formation of some small aggregates was observed in H_2_O_2_-exposed cultures (Fig. S3A and B), indicating that members of the *Nitrosopumilus* genus potentially remodel their membrane and/or extracellular matrix, including cell-to-cell attachment properties, in response to H_2_O_2_. However, the response of secreted/membrane-bound proteins observed for the three AOA strains investigated here could also represent a result of the renewal of damaged proteins in close proximity to the H_2_O_2_ production site (further discussed in Text S1 in the supplemental material).

10.1128/mSystems.00181-19.4 FIG S3 (A and B) Cell aggregation of Nitrosopumilus adriaticus NF5 observed during growth in medium without hydrogen peroxide (H_2_O_2_) scavengers. (C and D) CARD-FISH images of Nitrosopumilus adriaticus NF5 (DAPI [4′,6-diamidino-2-phenylindole]; blue) grown in coculture with Oceanicaulis alexandrii (bacterial EUBI probe; green). Download FIG S3, PDF file, 2.7 MB. Copyright © 2019 Bayer et al. 2019 Bayer et al. This content is distributed under the terms of the Creative Commons Attribution 4.0 International license.

Furthermore, a putative membrane-bound copper transport protein was detected at significantly higher relative abundance in the proteomes of the three *Nitrosopumilus* strains grown under conditions of exposure to H_2_O_2_ than in the proteomes derived from cultures containing catalase or O. alexandrii (Fig. 3). The N-terminal side of this protein exhibits homology with CopC family proteins, which are periplasmic copper binding proteins suggested to primarily play a role in bacterial copper homeostasis (64). E. coli cells containing excess copper were shown to be less sensitive to H_2_O_2_-induced DNA damage (65). Furthermore, reactions of Cu(I) and Cu(II) with H_2_O_2_ have recently been suggested to be involved in the formation of Cu(III) and O_2_^−^, respectively, instead of OH· (66). Hence, while classical Fenton reactions might induce oxidative damage and inactivation of iron-containing enzymes (67), copper import could represent a strategy to reduce or even prevent damage to macromolecules induced by OH·. Nevertheless, the function of this protein in AOA remains to be confirmed and requires further investigations.

### Metabolic interactions between *Nitrosopumilus* and Oceanicaulis alexandrii.

The peak concentrations of H_2_O_2_ in cocultures of the heterotrophic alphaproteobacterium Oceanicaulis alexandrii with *Nitrosopumilus* strains were on average 2 to 3 times lower than those measured in axenic *Nitrosopumilus* cultures (Fig. 1D to F), suggesting that O. alexandrii is capable of reducing the H_2_O_2_ concentration in AOA cultures. The initial description of O. alexandrii noted that strains of this bacterium are catalase positive (68), and, accordingly, the genome of the type strain O. alexandrii DSM 11625^T^ encodes two homologs of heme-containing catalase peroxidases belonging to the class I catalases (69). Amino acid residues forming the catalytic site of biochemically characterized heme-containing catalases are conserved in catalase homologs of O. alexandrii (Data Set S2B), suggesting that these homologs are bona fide catalases. One of these two homologs is characterized by an N-terminal signal peptide (Data Set S2B), suggesting that it is addressed to the periplasm and might be further secreted into the culture medium. Additionally, we detected proteotypic peptides of these catalase homologs in protein extracts prepared from cocultures of O. alexandrii and *Nitrosopumilus* spp., further suggesting that reduced concentrations of H_2_O_2_ in cocultures of O. alexandrii and AOA could result from O. alexandrii catalase activity.

10.1128/mSystems.00181-19.10 DATA SET S2 (A) Catalase-containing contigs of thaumarchaeal single-cell genomes AB-661-L21 and AB-661-M19 from Luo et al. (42) (catalases are marked in yellow). (B) Multiple-sequence alignment of class I catalase peroxidases, including sequences from Oceanicaulis alexandrii (blue) and sequences with known three-dimensional (3D) structures obtained from PeroxiBase. Color scheme: black = 100% sequence similarity; gray = 90% to 99% sequence similarity. Signal peptides are indicated in red. Essential residues involved in catalysis are labeled with an asterisk. Download Data Set S2, DOCX file, 0.2 MB. Copyright © 2019 Bayer et al. 2019 Bayer et al. This content is distributed under the terms of the Creative Commons Attribution 4.0 International license.

**FIG 4:**
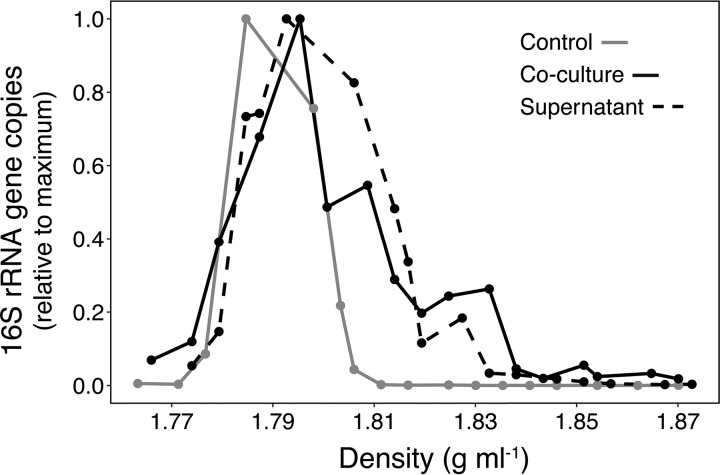
Proportion of O. alexandrii 16S rRNA gene copies recovered from RNA-SIP gradient fractions. O. alexandrii was grown in coculture with N. piranensis in medium containing ^13^C-labeled bicarbonate (solid black line) and on N. piranensis supernatant (dashed black line). Growth in medium containing unlabeled yeast extract-peptone and ^13^C-labeled bicarbonate served as a control (solid gray line).

O. alexandrii was able to grow in coculture with all three *Nitrosopumilus* strains, as well as in the supernatant of *Nitrosopumilus* cultures (Fig. S4A; see also Table S1). RNA stable isotope probing (RNA-SIP) was used to directly confirm the transfer of organic carbon from autotrophic *Nitrosopumilus* cells to O. alexandrii. After two consecutive passages in medium containing ^13^C-labeled bicarbonate, 16S rRNA sequences of N. piranensis showed a clear enrichment in the “heavy” (^13^C) fraction (approximately 25% of the RNA was labeled) relative to control incubations (Fig. S5). When O. alexandrii was subsequently grown in coculture with N. piranensis cells or in N. piranensis culture supernatant, 16S rRNA sequences of O. alexandrii showed an enrichment in the “heavy” (^13^C) fraction (approximately 17% in coculture and 8% on supernatant) compared to the control incubations (Fig. 4). Furthermore, no incorporation of ^13^C directly from bicarbonate via anaplerotic reactions was observed in the control treatment (i.e., O. alexandrii cells growing in yeast extract-peptone medium supplemented with ^13^C-labeled bicarbonate) (Fig. 4).

10.1128/mSystems.00181-19.5 FIG S4 (A) Growth curves of Oceanicaulis alexandrii and Nitrosopumilus adriaticus NF5 in coculture with addition of catalase. (B) Growth of O. alexandrii on culture supernatant of three *Nitrosopumilus* strains (N. adriaticus NF5, N. piranensis D3C, and N. maritimus SCM1) and in medium without the addition of any carbon source (Control). Error bars represent standard deviations of measurements from triplicate cultures. Download FIG S4, PDF file, 0.3 MB. Copyright © 2019 Bayer et al. 2019 Bayer et al. This content is distributed under the terms of the Creative Commons Attribution 4.0 International license.

10.1128/mSystems.00181-19.6 FIG S5 Proportion of N. piranensis D3C 16S rRNA gene copies recovered from RNA-SIP gradient fractions under conditions of growth in medium containing unlabeled bicarbonate (gray line) and after two passages into medium containing ^13^C-labeled bicarbonate (black line). Download FIG S5, PDF file, 0.2 MB. Copyright © 2019 Bayer et al. 2019 Bayer et al. This content is distributed under the terms of the Creative Commons Attribution 4.0 International license.

While the ^13^C enrichment clearly confirms the incorporation of *Nitrosopumilus*-derived organic carbon into the biomass of O. alexandrii, these results do not clarify the identity of organic carbon compounds that mediate the metabolic interaction between AOA and O. alexandrii. The alphaproteobacterium generally showed the highest growth rates during exponential growth of the AOA strains (Table S1; see also Fig. S4B), indicating growth on compounds released by active *Nitrosopumilus* cells rather than subsistence on dead cell material. We showed in another study that all investigated strains indeed released organic matter, including labile compounds such as amino acids and thymidine (70). However, the possibility of proteolytic growth on *Nitrosopumilus* cells cannot be completely excluded. Nevertheless, growth on released, soluble substances is also supported by the ability of O. alexandrii to grow on *Nitrosopumilus* culture supernatant, suggesting a rather unspecific interaction. And yet, O. alexandrii cells often appeared to be attached to *Nitrosopumilus* cells during growth in coculture (Fig. S3C and D). The highest growth yields of O. alexandrii were observed in *Nitrosopumilus* cocultures with added catalase (Table S1), suggesting either a higher level of release of organic matter by *Nitrosopumilus* under optimal conditions or growth of O. alexandrii on the purified catalase itself. Alternatively, the presence of a purified catalase could provide a growth advantage to O. alexandrii by reducing the amount of catalase it needs to produce. Furthermore, O. alexandrii achieved higher cell abundances in coculture with N. piranensis (composing up to 25% of the cells during the incubation period, Table S1) than were seen with N. adriaticus or N. maritimus. Hence, the levels of quantity and/or quality of organic carbon released by different *Nitrosopumilus* strains potentially differ under the same culture conditions.

### Conclusions.

Our results, combined with the results of previous studies, suggest that sensitivity to H_2_O_2_ is common among different members of the *Nitrosopumilus* genus and contribute to the understanding of the physiological and molecular responses of AOA to H_2_O_2_. The extent of the sensitivities of marine AOA to H_2_O_2_ appears to differ between strains, which may lead to niche differentiation.

The absence and/or loss of a specific function (i.e., H_2_O_2_ detoxification) has been suggested to provide a selective advantage by conserving an organism’s limiting resources (71). The ocean’s most abundant free-living prokaryotes, including *Prochlorococcu*s, “*Candidatus* Pelagibacter” (SAR11 clade), and AOA, can grow axenically only when such missing metabolic functions are provided (14, 33, 72). Our results suggest that marine AOA rely on H_2_O_2_ detoxification during periods of high activity and release organic compounds, thereby attracting heterotrophic prokaryotes that provide the missing catalase function.

Interactions between *Nitrosopumilus* spp. and the alphaproteobacterium O. alexandrii are reminiscent of interactions between heterotrophic bacteria and phytoplankton cells (i.e., within the “phycosphere”), and the importance of these microscale interactions for aquatic ecosystems is widely acknowledged (73). Similarly, metabolic interactions within the immediate surroundings of AOA cells might represent a successful ecological strategy for heterotrophic bacteria, especially in locations below the euphotic layer of the ocean. Microbial radiocarbon signatures indicate that chemolithoautotrophic production can supply up to 95% of the organic carbon incorporated by free-living microbial communities in mesopelagic waters (74). AOA are the most abundant chemolithoautotrophic microbes in the global ocean, suggesting that they could play a crucial role in the production of reduced carbon compounds from inorganic carbon and therefore in the provision of labile organic matter for heterotrophic prokaryotes.

## MATERIALS AND METHODS

### Cultivation procedures and H_2_O_2_ sensitivity experiments.

Axenic cultures of Nitrosopumilus adriaticus NF5, Nitrosopumilus piranensis D3C, and Nitrosopumilus maritimus SCM1 were routinely grown in synthetic *Crenarchaeota* medium (SCM) in the dark as previously described (27, 75) with the addition of catalase (Sigma catalog no. C1345) (5 units ml^−1^ final concentration). Cultures were maintained in 30-ml polypropylene plastic bottles, and growth was monitored via flow cytometry (described in Text S1 in the supplemental material) and by measuring nitrite production levels (76).

Prior to establishing different culture treatments, *Nitrosopumilus* cultures were grown without the addition of catalase for one passage (initial cell abundances in the preculture were ≥7 × 10^6^ ml^−1^) to ensure exclusion of the remaining catalase activity and catalase carryover to the culture medium. Subsequently, each AOA strain was grown under four distinct sets of conditions and triplicate cultures were prepared for each growth condition and strain. The tested growth conditions were as follows: (i) no H_2_O_2_ scavenger, (ii) supplementation with catalase, (iii) inoculation with Oceanicaulis alexandrii, and (iiv) both inoculation with O. alexandrii and supplementation with catalase. To establish cocultures, O. alexandrii was grown in SCM medium with 0.01% yeast extract-peptone and cells were harvested via centrifugation (10,000 × *g*, 10°C, 15 min) after 3 days, washed three times with SCM culture medium, and added to freshly inoculated *Nitrosopumilus* cultures (5% O. alexandrii and 95% *Nitrosopumilus* spp. [based on cell abundance measurements]). Catalyzed reporter deposition-fluorescence *in situ* hybridization (CARD-FISH) was performed on cocultures to differentiate between bacterial cell abundance and archaeal cell abundance (described in Text S1 in the supplemental material).

Furthermore, the effect of the AOA inoculum size on their cellular response to H_2_O_2_ was tested by establishing duplicate cultures of *Nitrosopumilus* spp. with various initial cell abundances (∼2 × 10^5^, ∼8 × 10^5^, ∼3 × 10^6^, and 7 × 10^6^ cells ml^−1^) without catalase addition. H_2_O_2_ concentrations were measured with a fluorescence-based assay (Sigma-Aldrich, catalog no. MAK165) according to the manufacturer’s protocol.

### Proteomics and differential protein expression analysis.

Triplicate cultures of *Nitrosopumilus* spp. were grown in 250-ml Schott bottles for each of the following treatments: with addition of catalase, in coculture with Oceanicaulis alexandrii, without catalase and O. alexandrii (H_2_O_2_ inhibited), and without catalase and O. alexandrii at a high (7 × 10^6^ cells ml^−1^) initial cell abundance (H_2_O_2_ noninhibited). Culture conditions were established as described in the section above. Cells were harvested during exponential growth via centrifugation (18,500 × *g*, 4°C, 1.5 h), and cell pellets were immediately frozen at −80°C until whole-cell protein extraction was performed.

Proteins were extracted from cell pellets and subjected to denaturing polyacrylamide gel electrophoresis (SDS-PAGE) followed by overnight trypsin in-gel digestion (described in detail in Text S1 in the supplemental material). Desalted peptides were resuspended in an aqueous solution containing 2% acetonitrile and 0.1% formic acid to a concentration of 0.2 μg μl^−1^ (1 μg total) prior to loading onto an Easy-spray column (Thermo Fisher Scientific PepMap RSCL) (C_18_; 500 mm by 75 μm; pore size of 2.0 μm). Peptides were separated during a 270-min gradient step using a flow rate of 300 nl min^−1^ and a one-dimensional (1D) nano-LC instrument (Dionex UltiMate 3000; Thermo Fisher Scientific) coupled to an Orbitrap Elite mass spectrometer (Thermo Fisher Scientific, Bremen, Germany) (see Text S1 in the supplemental material). Each of the 36 protein extracts was analyzed twice (resulting in a total of 72 proteomic profiles), and the two technical replicates were combined for bioinformatic analysis (resulting in 36 combined proteomes). Acquired MS/MS spectra were analyzed using the SEQUEST-HT algorithm implemented in Proteome Discoverer 2.2 software (Thermo Fisher Scientific), and spectra were searched against the entire set of translated coding sequences of Nitrosopumilus adriaticus NF5 (2627854092), Nitrosopumilus piranensis D3C (2627853696), and Nitrosopumilus maritimus SCM1 (641228499), downloaded from the Integrated Microbial Genomes (IMG) database (77). Protein matches were accepted if they were identified by at least one unique peptide and with high confidence (details can be found in Text S1 in the supplemental material), and proteins were quantified using the normalized spectral abundance factor (NSAF) approach (78) as follows:

$${\text{NSAF}}_{k}={\left(\frac{\text{PSM}}{L}\right)}_{k}/\overset{N}{\underset{i=1}{\sum }}{\left(\frac{\text{PSM}}{L}\right)}_{i}$$

where the total number of spectral counts for the matching peptides from protein *k* (PSM) was divided by the protein length (*L*) and then divided by the sum of PSM/*L* for all *N* proteins.

In order to adequately match genes shared by the three *Nitrosopumilus* strains and subsequently compare their individual proteomic responses, orthologous groups (OGs) were constructed on the basis of their entire set of coding sequences using OrthoFinder (version 1.0.8) with standard settings (79). The complete list of assigned OGs and their annotations can be found in Data Set S1A and B in the supplemental material. Differential levels of expression of proteins recovered for each strain and treatment were tested with the DESeq2 Bioconductor package (version 1.20.0) (80) in the R software environment (version 3.5.0) using default parameters and spectral counts as input data based on the recommendations of Langley and Mayr (81). All possible (i.e., all six) pairwise comparisons between the four different treatments, (i.e., with catalase, with O. alexandrii, H_2_O_2_ inhibited, and H_2_O_2_ noninhibited) were performed separately for each *Nitrosopumilus* strain. Each test included three biological replicates per treatment, with the exception of the N. maritimus SCM1 “H_2_O_2_ inhibited” treatment, where one biological replicate was excluded from all analyses because of apparent problems during MS analyses that resulted in poor identification of the proteins. Probability values (*P* values) were adjusted using the Benjamini-Hochberg correction method as previously described (80, 81). The following filter criteria were applied in DESeq2: adjusted *P* value, <0.05; log 2-fold difference between treatments: greater than or equal to 2 and less than or equal to −2; mean of normalized counts, ≥3. Proteins that showed significant pairwise correlations were visualized with the pheatmap package (version 1.0.12) (82) in the R software environment (83). Columns and rows were clustered based on Euclidean distances corresponding to differences between treatments and relative protein abundances, respectively, as implemented in the pheatmap package. Curation of the annotations of proteins showing significant changes in relative abundance was performed by sequence similarity searches using BLAST (84) and the RefSeq (release 92) and UniprotKB/Swissprot (release 2019_01) databases (85, 86), and protein domain searches were performed using InterProScan (release 72.0) (87). Signal peptides were identified with PRED-Signal (88) and SignalP5.0 (89) to determine if proteins were potentially addressed to the membrane and/or released to the (pseudo)periplasmic space, and additional homology modeling of proteins and functional predictions were carried out with Phyre2 (90).

### ^13^C-RNA-stable isotope probing (^13^C-RNA-SIP).

The three AOA strains investigated in this study were grown in SCM medium containing ^13^C-labeled bicarbonate (2 mM final concentration) for two consecutive passages, each lasting for 5 to 7 days. Subsequently, O. alexandrii was grown with each AOA in separate cocultures and axenically on the culture supernatant of the three AOA strains. Cocultures were established as described above in the cultivation procedure section. Culture supernatants were obtained via centrifugation (10,000 × *g*, 10°C, 30 min) and gentle serial filtration through 0.2-μm-pore-size filters (Durapore, Millipore; 47 mm) and 0.1-μm-pore-size filters (Durapore, Millipore; 33 mm). Cells of O. alexandrii grown in yeast extract-peptone medium containing ^13^C-labeled bicarbonate (2 mM) served as a control to evaluate potential labeling of O. alexandrii rRNA via anaplerotic reactions. After 5 days of incubation, cultures were harvested by filtration through 0.2-μm-pore-size polycarbonate filters (Millipore; 47 mm) which were immediately frozen at −80°C. RNA was extracted according to the protocol of Angel (91) with some modifications for the use of filters (described in detail in Text S1 in the supplemental material), and samples of late-exponential-phase cultures of N. piranensis were selected for tracing the incorporation of AOA-derived organic matter into O. alexandrii 16S rRNA.

Subsequently, heavy (^13^C-labeled) RNA was separated from light (“natural” ^13^C/^12^C isotope ratio) RNA by isopycnic centrifugation. Approximately 300 ng of RNA was mixed with cesium trifluoroacetate (CsTFA; GE Healthcare), HiDi formamide (Thermo Fisher Scientific), and gradient buffer (0.1 M Tris-HCl [pH 8.0], 0.1 M KCl, 1 mM EDTA) as described previously (92). Samples were centrifuged at 130,000 × *g* at 20°C for at least 65 h in an Optima L-100 XP ultracentrifuge with a VTi 90 rotor (Beckman Coulter), and the resulting CsTFA density gradients were fractionated into 20 equal (250-μl) fractions. Fractions accounting for densities ranging between 1.760 and 1.875 g ml^−1^ were used for downstream analysis. RNA was precipitated at −80°C after addition of 2.5 volumes of 100% ethanol, 0.5 volumes of 5 M NH_4_-acetate, and 2 μl glycogen (molecular biology grade; Thermo Fisher Scientific) and pelleted by centrifugation at 20,000 × *g* for 30 min at 4°C. The RNA pellets were washed with ice-cold 75% ethanol, air-dried, and subsequently resuspended in 10 μl RNA storage solution (Ambion). cDNA was synthesized using SuperScript III reverse transcriptase and random hexamer primers (both from Thermo Fisher Scientific) according to the manufacturer’s protocol. 16S rRNA copies from individual SIP fraction were quantified by quantitative PCR (qPCR) (Bio-Rad) (see Text S1 in the supplemental material), and results are expressed as a proportion of the total number of 16S rRNA copies from all SIP fractions.

### Data availability.

All acquired raw spectrum files and proteomic result files, including identified peptides, relative protein abundances, and DESeq outputs, are available on MassIVE (https://massive.ucsd.edu) under accession number MSV000083517 (ftp://massive.ucsd.edu/MSV000083517).
