# Supplementary material for: Proteomic Response of Three Marine Ammonia-Oxidizing Archaea to Hydrogen Peroxide and Their Metabolic Interactions with a Heterotrophic Alphaproteobacterium
Source: mSystems. 2019 Jun 25;4(4):e00181-19. doi: 10.1128/mSystems.00181-19 (PMC6593220; doi:10.1128/mSystems.00181-19)
Supplement: TABLE S1 [file mSystems.00181-19-st001.docx]

| **Species** | **Treatment** | **Time (d)** | **Archaea (mL^-1^)** | **Bacteria (mL^-1^)** | **Bacteria (%)** |
| --- | --- | --- | --- | --- | --- |
| *N. adriaticus* | none | 0 | 3.10E+06 |  |  |
| *N. adriaticus* | none | 3 | 6.39E+06 |  |  |
| *N. adriaticus* | none | 5 | 6.76E+06 |  |  |
| *N. adriaticus* | none | 7 | 6.43E+06 |  |  |
| *N. adriaticus* | none | 9 | 6.80E+06 |  |  |
| *N. adriaticus* | none | 13 | 6.79E+06 |  |  |
| *N. adriaticus* | none | 25 | 4.35E+06 |  |  |
| *N. adriaticus* | CAT | 0 | 3.10E+06 |  |  |
| *N. adriaticus* | CAT | 3 | 1.07E+07 |  |  |
| *N. adriaticus* | CAT | 5 | 2.35E+07 |  |  |
| *N. adriaticus* | CAT | 7 | 3.11E+07 |  |  |
| *N. adriaticus* | CAT | 9 | 3.46E+07 |  |  |
| *N. adriaticus* | CAT | 11 | 3.67E+07 |  |  |
| *N. adriaticus* | co-culture | 0 | 3.14E+06 | 1.57E+05 | 5 |
| *N. adriaticus* | co-culture | 3 | 8.20E+06 | 4.51E+05 | 5.5 |
| *N. adriaticus* | co-culture | 5 | 1.28E+07 | 8.73E+05 | 6.8 |
| *N. adriaticus* | co-culture | 7 | 1.38E+07 | 4.54E+05 | 3.3 |
| *N. adriaticus* | co-culture | 9 | 1.84E+07 | 5.17E+05 | 2.8 |
| *N. adriaticus* | co-culture | 11 | 2.29E+07 | 5.03E+05 | 2.2 |
| *N. adriaticus* | co-culture | 13 | 3.49E+07 | 5.23E+05 | 1.5 |
| *N. adriaticus* | co-culture + CAT | 0 | 3.00E+06 | 1.50E+05 | 5 |
| *N. adriaticus* | co-culture + CAT | 3 | 1.19E+07 | 2.26E+05 | 1.9 |
| *N. adriaticus* | co-culture + CAT | 5 | 2.67E+07 | 1.57E+06 | 5.9 |
| *N. adriaticus* | co-culture + CAT | 7 | 3.03E+07 | 1.61E+06 | 5.3 |
| *N. adriaticus* | co-culture + CAT | 9 | 3.47E+07 | 1.74E+06 | 5 |
| *N. adriaticus* | co-culture + CAT | 11 | 3.35E+07 | 1.41E+06 | 4.2 |
| *N. piranensis* | none | 0 | 2.05E+06 |  |  |
| *N. piranensis* | none | 6 | 4.38E+06 |  |  |
| *N. piranensis* | none | 9 | 4.34E+06 |  |  |
| *N. piranensis* | none | 17 | 3.67E+06 |  |  |
| *N. piranensis* | none | 25 | 3.48E+06 |  |  |
| *N. piranensis* | CAT | 0 | 2.05E+06 |  |  |
| *N. piranensis* | CAT | 6 | 1.17E+07 |  |  |
| *N. piranensis* | CAT | 9 | 2.94E+07 |  |  |
| *N. piranensis* | co-culture | 0 | 2.16E+06 | 1.08E+05 | 5 |
| *N. piranensis* | co-culture | 6 | 8.22E+06 | 8.14E+05 | 9.9 |
| *N. piranensis* | co-culture | 9 | 1.04E+07 | 3.85E+05 | 3.7 |
| *N. piranensis* | co-culture | 13 | 3.24E+07 | 1.78E+06 | 5.5 |
| *N. piranensis* | co-culture + CAT | 0 | 2.20E+06 | 1.10E+05 | 5 |
| *N. piranensis* | co-culture + CAT | 6 | 1.18E+07 | 2.96E+06 | 25.1 |
| *N. piranensis* | co-culture + CAT | 9 | 2.94E+07 | 3.73E+06 | 12.7 |
| *N. maritimus* | none | 0 | 1.84E+06 |  |  |
| *N. maritimus* | none | 6 | 1.05E+07 |  |  |
| *N. maritimus* | none | 9 | 1.04E+07 |  |  |
| *N. maritimus* | none | 14 | 1.19E+07 |  |  |
| *N. maritimus* | none | 17 | 1.08E+07 |  |  |
| *N. maritimus* | none | 25 | 8.56E+06 |  |  |
| *N. maritimus* | CAT | 0 | 1.84E+06 |  |  |
| *N. maritimus* | CAT | 6 | 2.94E+07 |  |  |
| *N. maritimus* | CAT | 9 | 4.82E+07 |  |  |
| *N. maritimus* | co-culture | 0 | 1.99E+06 | 9.97E+04 | 5 |
| *N. maritimus* | co-culture | 6 | 1.81E+07 | 5.07E+05 | 2.8 |
| *N. maritimus* | co-culture | 8 | 2.53E+07 | 7.84E+05 | 3.1 |
| *N. maritimus* | co-culture + CAT | 0 | 2.00E+06 | 1.00E+05 | 5 |
| *N. maritimus* | co-culture + CAT | 6 | 3.52E+07 | 9.16E+05 | 2.6 |
| *N. maritimus* | co-culture + CAT | 8 | 5.31E+07 | 1.70E+06 | 3.2 |
