# Supplementary figures and images for: Proteomic Response of Three Marine Ammonia-Oxidizing Archaea to Hydrogen Peroxide and Their Metabolic Interactions with a Heterotrophic Alphaproteobacterium
Source: mSystems. 2019 Jun 25;4(4):e00181-19. doi: 10.1128/mSystems.00181-19 (PMC6593220; doi:10.1128/mSystems.00181-19)

**A**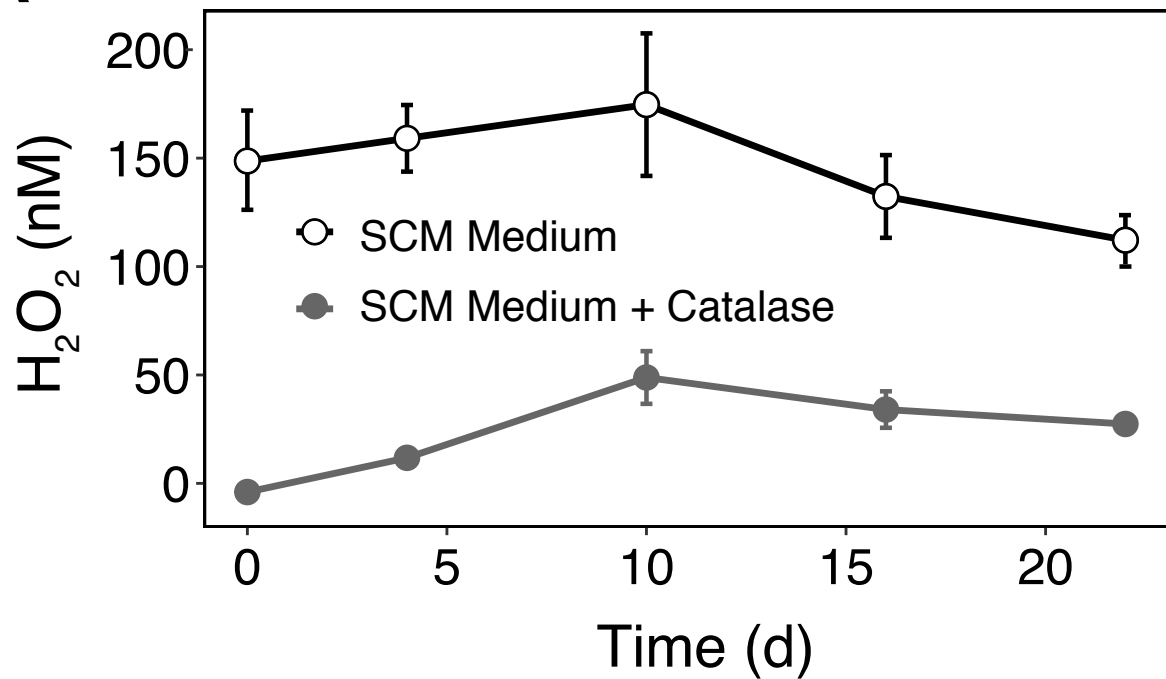**B**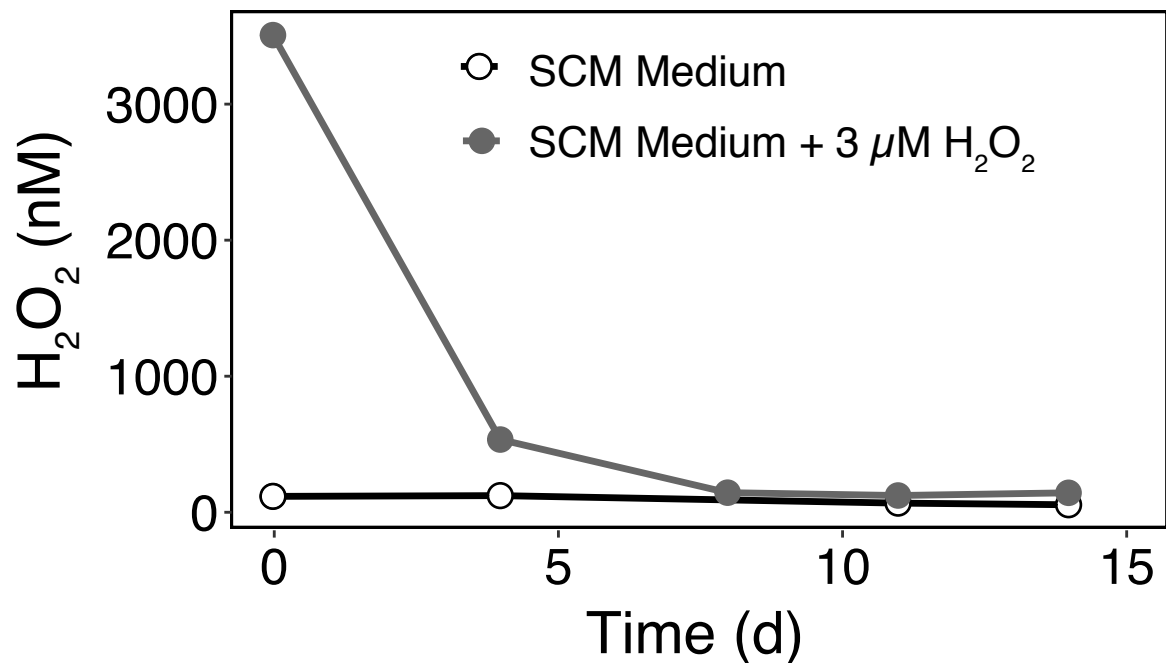

Supplement: FIG S1 [file mSystems.00181-19-sf001.pdf]

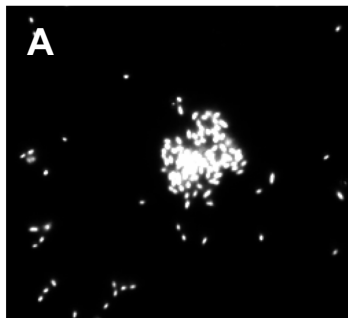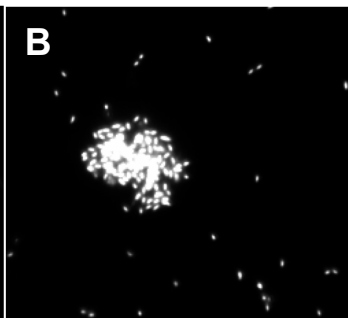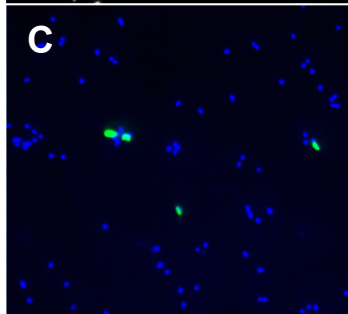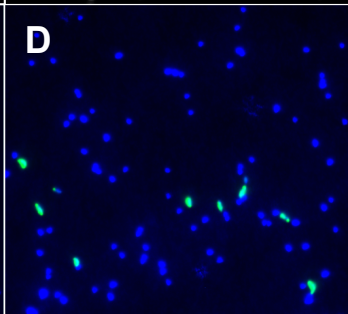

Supplement: FIG S3 [file mSystems.00181-19-sf003.pdf]

**A**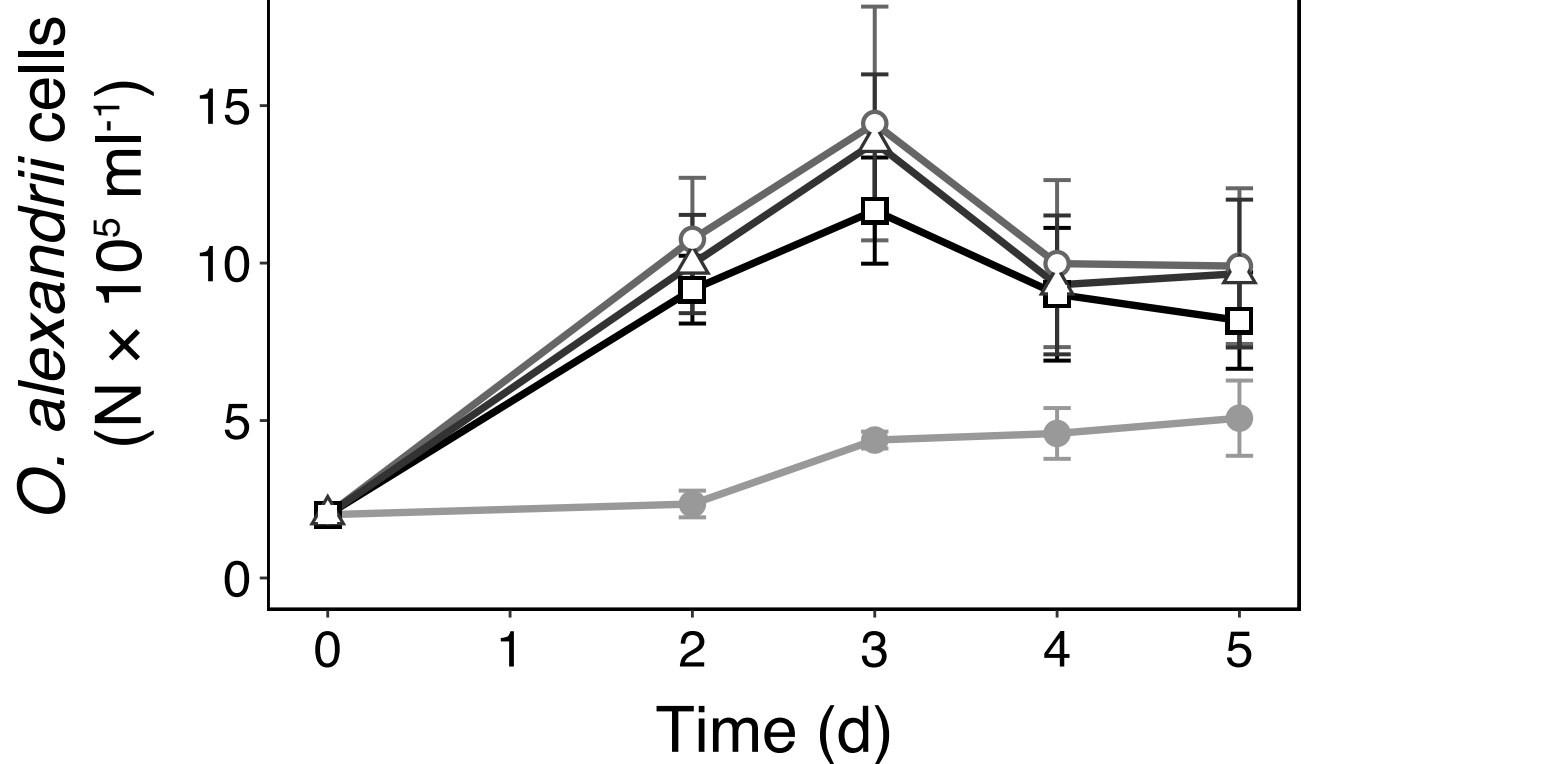**B**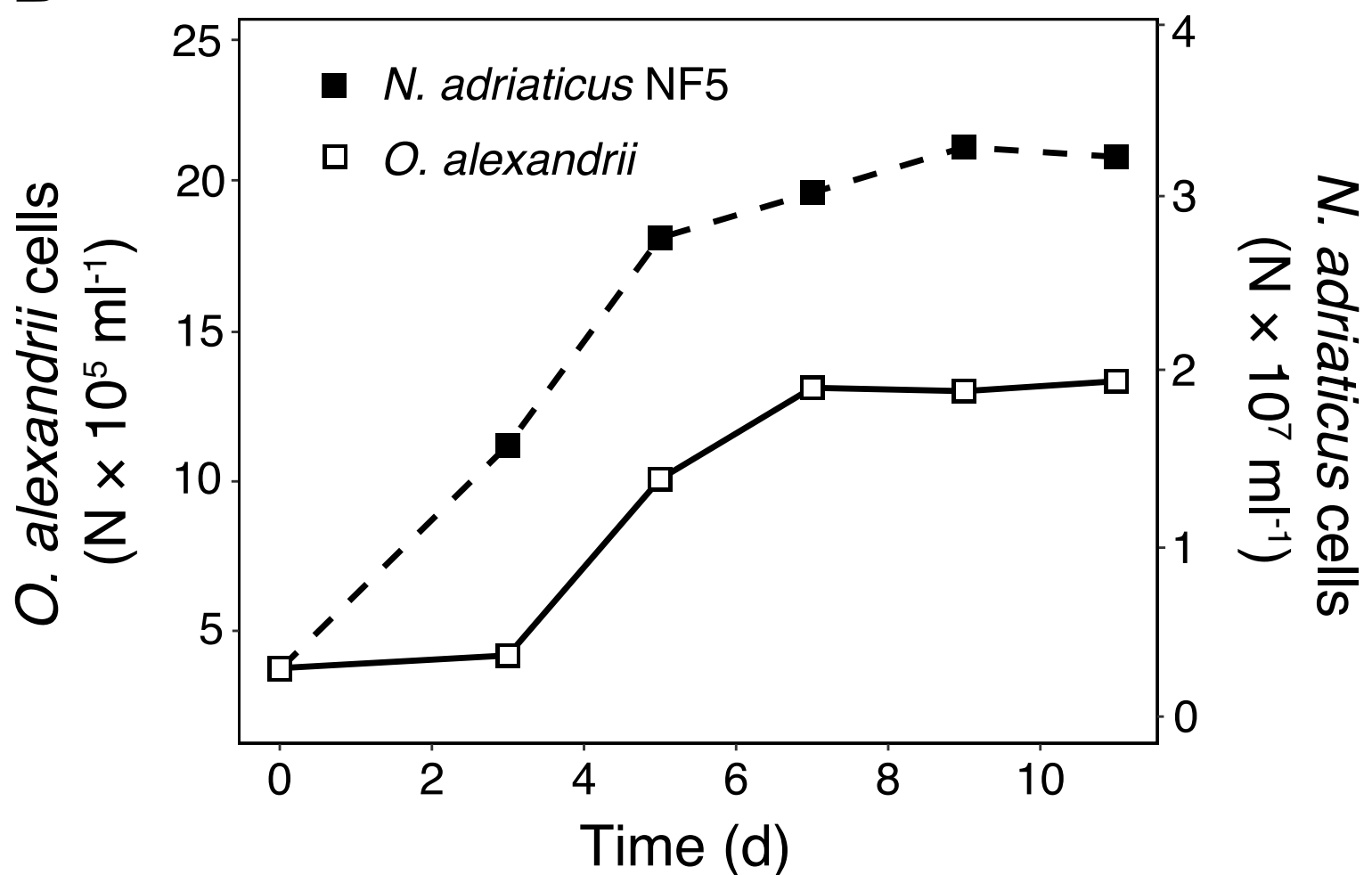

Supplement: FIG S4 [file mSystems.00181-19-sf004.pdf]

16S rRNA gene copies  
(relative to maximum)

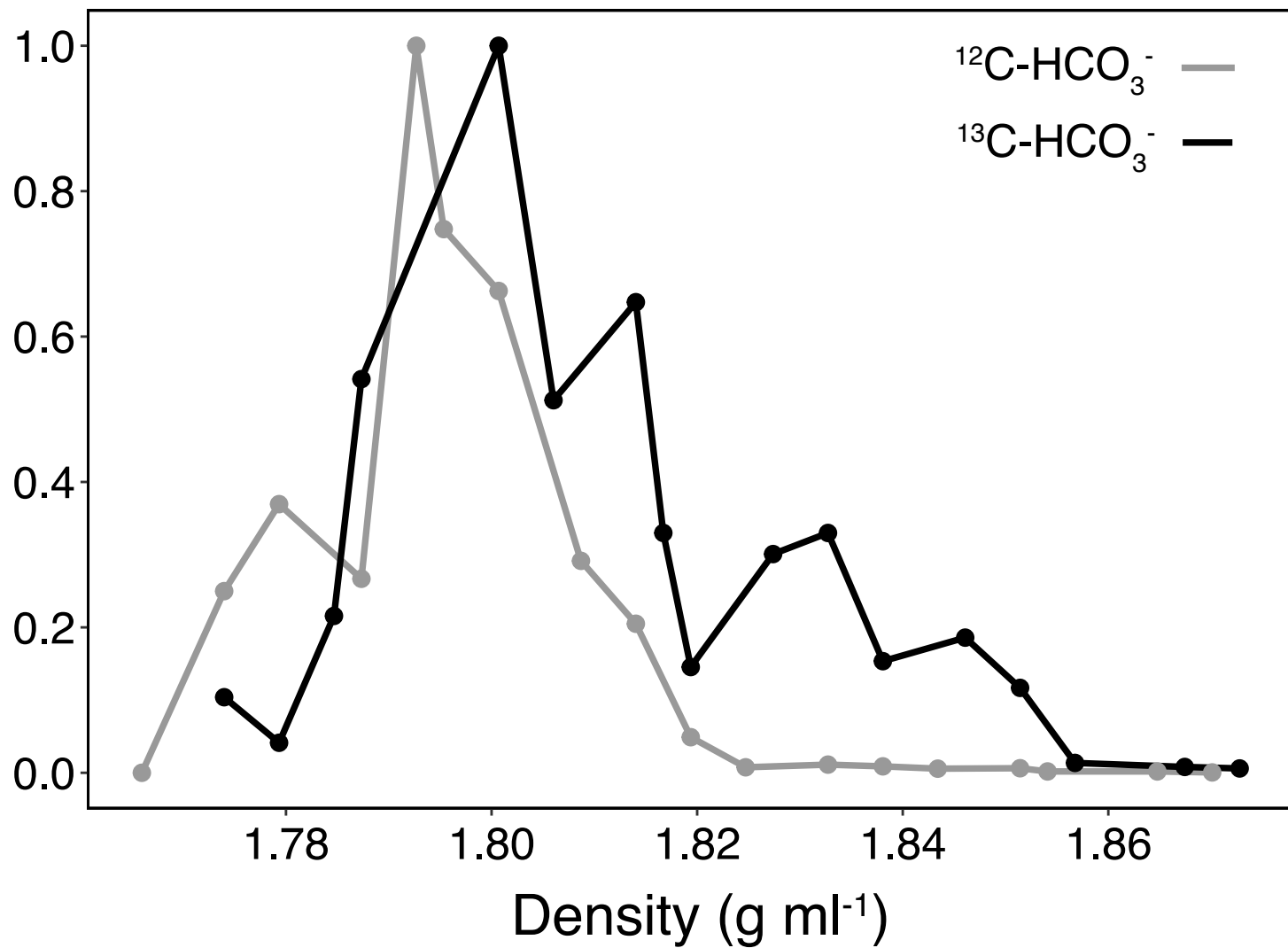

Supplement: FIG S5 [file mSystems.00181-19-sf005.pdf]
