# Supplementary material for: Proteomic Response of Three Marine Ammonia-Oxidizing Archaea to Hydrogen Peroxide and Their Metabolic Interactions with a Heterotrophic Alphaproteobacterium
Source: mSystems. 2019 Jun 25;4(4):e00181-19. doi: 10.1128/mSystems.00181-19 (PMC6593220; doi:10.1128/mSystems.00181-19)
Supplement: TABLE S2 [file mSystems.00181-19-st002.docx]

**A**

| **Treatment** | ***N. adriaticus* NF5** | ***N. piranensis* D3C** | ***N. maritimus* SCM1** | *Shared* |
| --- | --- | --- | --- | --- |
| Catalase | 1372 | 1326 | 1259 | 900 |
| *O. alexandrii* | 1301 | 1272 | 1228 | 806 |
| H_2_O_2_ non-inhibited | 1235 | 1245 | 1020 | 765 |
| H_2_O_2_ inhibited | 1236 | 1194 | 1174 | 698 |
| *Shared* | 1040 | 1027 | 856 |  |

**B**

|  | | | ***N. adriaticus* NF5** | | ***N. piranensis* D3C** | | ***N. maritimus* SCM1** | |  |
| --- | --- | --- | --- | --- | --- | --- | --- | --- | --- |
| Treatment 1 | *vs* | Treatment 2 | Proteins | % of total | Proteins | % of total | Proteins | % of total | *shared* |
| Catalase |  | H_2_O_2_ non-inhibited | 68 | 4.96 | 70 | 5.28 | 60 | 4.77 | 33 |
| Catalase |  | H_2_O_2_ inhibited | 73 | 5.32 | 86 | 6.49 | 56 | 4.45 | 33 |
| *O. alexandrii* |  | H_2_O_2_ non-inhibited | 80 | 6.15 | 88 | 6.92 | 56 | 4.56 | 33 |
| *O. alexandrii* |  | H_2_O_2_ inhibited | 86 | 6.61 | 109 | 8.57 | 66 | 5.37 | 33 |
| H_2_O_2_ non-inhibited |  | H_2_O_2_ inhibited | 3 | 0.24 | 3 | 0.24 | 3 | 0.39 | 0 |
| *O. alexandrii* |  | Catalase | 20 | 1.54 | 12 | 0.94 | 12 | 0.98 | 5 |
