# Supplementary material for: Proteomic Response of Three Marine Ammonia-Oxidizing Archaea to Hydrogen Peroxide and Their Metabolic Interactions with a Heterotrophic Alphaproteobacterium
Source: mSystems. 2019 Jun 25;4(4):e00181-19. doi: 10.1128/mSystems.00181-19 (PMC6593220; doi:10.1128/mSystems.00181-19)
Supplement: TEXT S1 [file mSystems.00181-19-s0001.docx]

**Supplementary Information (SI)**

**Supplementary Material and Methods**

**Protein extraction**

Proteins were extracted from cell pellets by adding 500 µL lysis buffer (100 mM Tris-HCl pH 7.5, 150 mM NaCl, 1 mM DTT, 1 % SDS, 10 mM EDTA) followed by three freeze-thaw cycles and 5 cycles of sonication with a sonication probe (Sonopuls HD 2070, Bandelin) for 30 sec intervals at 20% intensity. Cell lysates were centrifuged at 20 000 *g* at 4°C for 10 min and the supernatant was transferred into a new tube. Proteins were co-precipitated with 0.015% deoxycholate and 6% trichloroacetic acid (TCA) on ice for 1h and washed once with ice-cold acetone according to the protocol of Bensadoun and Weinstein (1). Dried protein pellets were resuspended in 50 µL of 8 M urea containing 4% SDS and protein concentrations were measured with the bicinchoninic acid (BCA) assay using BSA as a standard. 20-50 µg of protein were subjected to denaturing polyacrylamide gel-electrophoresis (SDS-PAGE), stained with Commassie staining solution and de-stained in 40% (v/v) methanol containing 2% (v/v) acetic acid as described in Valledor and Weckwerth (2). Gel bands were excised without fractionating the bands, cut into 1 x 1 mm pieces on a sterile glass plate and transferred into protein-low binding tubes (Eppendorf). Gel pieces were de-stained with 200 mM ammonium bicarbonate containing 50% acetonitrile at 37°C for 30 min and cysteines were reduced and alkylated with 10 mM DTT and 55 mM iodoacetoamide (IAA), respectively, as described by Shevchenko et al. (3) prior to overnight trypsin digestion according to Valledor and Weckwerth (2). Peptides were extracted and desalted using 96-well plates (Spec 96-Well C18, Agilent) (2) and then resuspended in 2 % acetonitrile containing 0.1 % formic acid to a concentration of 0.2 µg µL^-1^ prior to injection into a one-dimensional nanoflow LC–MS/MS.

**LC-MS/MS analysis and peptide identification**

Five µL of desalinated peptides were eluted using an Easy-spray PepMap RSLC column (ThermoFisher Scientific, C18, 500 mm x 75 µm, pore size 2.0 µm), during a 270-min gradient from 5 to 40% (v/v) acetonitrile and 0.1% (v/v) formic acid with a controlled flow rate of 300 nL min. MS analysis was performed on an Orbitrap Elite mass spectrometer (Thermo Fisher Scientific). Specific tune settings for the MS were set as follows: Mass resolution for precursor ion analysis in FTMS (Fourier transform mass spectrometry): 60,000; full-scan mode, mass window for precursor ion: 1 m/z; spray voltage: 1.9 kV; temperature of the heated transfer capillary: 275°C, covering the range 350–1,800 m/z, and cyclomethicone used as lock mass (m/z 371.101230). Each full MS scan was followed by 20 dependent MS/MS scans in the Ion Trap, using rapid mode with centroid data in which the 20 most abundant peptide molecular ions are dynamically selected. The dynamic exclusion window was set to 30 s and an exclusion list of 500 entries. Dependent fragmentations were performed in CID (collision-induced dissociation) mode, with a normalized collision energy of 35, iso width of 2.0, activation Q of 0.250, and activation time of 30 msec. Ions with a +1 or unidentified charge state in the full MS were excluded from MS/MS analysis.

In Proteome Discoverer 2.2 (Thermo Fisher Scientific), the mass tolerance was set to 5 ppm for precursor masses and 0.6 Da for the fragment masses. Cysteine carbamidomethylation was set as static-, methionine oxidation and protein *N*-terminal acetylation as dynamic modifications. A maximum false discovery of 1% was allowed for both peptide and protein levels. Trypsin was specified as the proteolytic enzyme and 2 missed cleavages were allowed with a maximum of 3 equal post-translational modifications (PTMs) per peptide. The obtained peptide spectrum matches (PSMs) were filtered with the Percolator tool based on a scoring of maximum delta Cn 0.05, a strict false discovery rate (FDR) of 0.01 with validation based on q-values.

**RNA extraction for Stable Isotope Probing (RNA-SIP)**

Tubes containing the filters were submerged in liquid nitrogen and ground into small pieces with a metal spatula. Phosphate buffer (750 µL, 120 mM, pH 5.5) and 250 µL TNS buffer (500 mM Tris base, 100 mM NaCl, 10% SDS) were added to the filter pieces, and cells were lysed with 5x freeze and thaw cycles. After centrifugation (20 000 *g*, 4°C, 3 min) the supernatant was added to a fresh tube and one volume of TE saturated phenol (pH 5.5) was added. RNA was purified using standard phenol/chloroform/isoamyl alcohol and chloroform/isoamyl alcohol purification, and the supernatant was transferred to non-stick RNase-free microfuge tubes (Eppendorf). Nucleic acids were precipitated with 30% polyethylene glycol (PEG) in 1.6 M NaCl and 2 μL glycogen (Thermo Scientific), washed once with ice-cold 75% EtOH and resuspended in low TE buffer. DNA was digested with TURBO DNase (Ambion) and purified using the RNeasy MinElute Cleanup Kit (Qiagen). Complete DNA removal was validated via PCR amplification.

**Quantitative PCR of SIP fractions**

The relative number of bacterial and archaeal rRNA copies were determined using the primers Oc-1369F 5’-CGG TGA ATA CGT TCC CGG-3’ and Oc-1492R 5’-GGC TAC CTT GTT ACG ACT T-3’ for *Oceanicaulis alexandrii* (modified from Smith et al (4)) and MCGI-391F 5’-AAG GTT ART CCG AGT GRT TTC-3’ and MCGI-554R 5’-TGA CCA CTT GAG GTG CTG-3’ for *Nitrosopumilus* spp. (5). The 20 µL qPCR reaction mix contained 1x IQTM SYBR Green Supermix (BIO-RAD), 0.25 µM of each primer and 1 µL RNA from single gradient fractions. The thermal cycling program consisted of 3 min at 95°C, 39 cycles of 95°C for 15 sec, 60°C for 30 sec and 72°C for 39 sec followed by a melting curve from 60°C to 95°C by increments of 0.5°C every 5 sec. Data were processed using iQ5 Optical System software (Bio-Rad).

**Flow Cytometry**

Samples of 250 µl were fixed with glutaraldehyde (0.5% final concentration) for 10 min and subsequently flash-frozen in liquid nitrogen and stored at -80°C. Prior to analysis, samples were diluted (1:10 or 1:50) in 0.2 µm filtered Tris-EDTA buffer (1M Tris-HCl, 0.1M EDTA, pH 8) and stained with SYBR Green I (1:20 000). Enumeration of cells was performed on a FACSAria II flow cytometer (BD Biosciences) as previously described (6).

**Catalyzed Reporter Deposition Fluorescence In Situ Hybridization (CARD-FISH)**

Samples from one out of three biological replicates per strain and treatment were fixed overnight with formaldehyde (2% final conc.) at 4°C and subsequently filtered onto 25 mm diameter polycarbonate filters with a pore size of 0.2 µm (Millipore, GTTP). CARD-FISH was carried out as described previously (7) with the following modifications: Cell wall permeabilization for Bacteria was achieved with a lysozyme treatment (10 mg mL^-1^ lysozyme, 0.1 M Tris-HCl, 0.05 M EDTA) at 37°C for 1 h. For permeabilization of thaumarchaeal cell membranes, filters were incubated in 0.1 M HCl for 1 min as previously described (8). The hybridization buffer contained 0.05% (v/v) Triton-X instead of 0.02% (v/v) SDS. Filters were hybridized with horseradish peroxidase (HRP)-labeled oligonucleotide probe mixtures using the CREN537 and EUBI probes (9) to target *Nitrosopumilus* spp. and *Oceanicaulis alexandrii* 16S rRNA gene sequences, respectively.

Signal amplification with Alexa488 labelled tyramides, mixed with amplification buffer (10% dextran sulfate, 2 M NaCl, 0.1% Blocking, PBS) in a ratio of 1:500, was carried out at 46°C for 15 min. After signal amplification, filters were washed in PBS-T (0.05% Triton X100) and Milli-Q water and dried prior to mounting in a mix of DAPI and antifading solutions (DAPI 2 µg mL^-1^, 0.5 μg mL^-1^ PBS, 1 μg mL^-1^ Vectashield, 5.5 μg mL^-1^ Citifluor) on glass slides. Slides were examined at 1250x magnification under an epifluorescence microscope (Axio Imager.M2, Zeiss) and the images were manually evaluated and counted.

**Multiple sequence alignment of Class I catalase-peroxidases**

Protein sequences of putative catalases from *O. alexandrii* were downloaded from the Reference sequence (RefSeq) database at NCBI (10) and protein sequences from catalases with known 3D structure were downloaded from PeroxiBase (11). Multiple sequence alignment of full-length protein sequences was performed with the Muscle algorithm implemented in the MEGA 6 package (12). Optimized parameters were gap open −2.9 gap extend 0, hydrophobicity multiplier 2, the maximum of alignment iterations was set to 1,000 and the clustering method was UPGMB for the first two iterations and Neighbor-Joining (NJ) for other iterations and minimal diagonal length 28, as previously described (13). Information on the presence of conserved catalytic residues was obtained from Zamocky *et al.* (13) and signal peptides were identified with SignalP 5.0 (14).

**Supplementary Results and Discussion**

**Potential extracellular matrix-associated proteins in *Nitrosopumilus***

Membrane-associated S-layer proteins, PEFG-CTERM domain-containing proteins and thrombospondin type 3-like repeat (TT3R) containing proteins were among the proteins that exhibited the highest increase in relative abundance in response to H_2_O_2_ (see main text). Protein homology modelling of one of the thrombospondin type 3-like repeat containing proteins revealed that the C-terminal domain was matching to the human thrombospondin-2 (569-722 nt, 32 % id) (15), whereas the N-terminal domain showed homology to the human cartilage oligomeric matrix protein (30-345 nt, 32 % id) (16) and to the bacterial OmpA protein of the gram-negative bacterium *Capnocytophaga gingivalis* (30-210 nt, 45% id), which is thus far the only crystallized TT3R repeat containing prokaryotic protein (17). TT3R motifs in general have been identified in a variety of bacterial proteins in addition to OmpA homologs and myxobacterial MtsB-E proteins, including glucosidases and flagellar motor protein MotB (17). In eukaryotes, thrombospondins are extracellular glycoproteins with adhesive properties involved in synthesis and remodeling of the extracellular matrix by binding to proteoglycans and other proteins including collagens, laminins, integrins and fibronectin (18, 19). Interestingly, we identified a protein containing a lamin-tail domain (OG1004) and a protein containing a collagen triple helix repeat domain (OG0954) at high abundances in all three *Nitrosopumilus* strains when exposed to H_2_O_2_ (Fig. 3). The collagen domain-containing protein showed highest similarity to collagen-like proteins of various *Bacillus* species and other spore-forming Firmicutes, where they were shown to be structural components of their exosporium filaments (20). In addition, a protein containing a fibronectin type III family domain and a cadherin-like domain (OG1650) were identified in *N. adriaticus* and *N. maritimus* (Fig. S2A-C).

Alanyl-tRNA synthetase was detected at high relative abundance in proteomes *N. adriaticus* and *N. piranensis* (Fig. S2A-C) upon exposure to H_2_O_2_. In addition to branched-chain amino acids, membrane spanning helixes typically also contain a high alanine content (21). The putative thrombospondins of all three species have a high alanine content (10%) which potentially requires higher expression of alanyl-tRNA-synthetase (~0.01% relative abundance in non-H_2_O_2_-exposed cultures *vs*. ~0.1% in H_2_O_2_-exposed cultures). All other tRNA-synthetases required for putative extracellular matrix associated proteins were identified at similar levels (~0.1% relative abundance) in all treatments.

**Additional proteomic response of three *Nitrosopumilus* strains to H_2_O_2_**

Pyruvate phosphate dikinase (PPDK) was among the proteins that exhibited the highest changes in abundance during H_2_O_2_ exposure, being particularly highly abundant in *N. maritimus* cultures (Fig. 3). PPDK is typically involved in gluconeogenesis, converting pyruvate into phosphoenolpyruvate (PEP) but can also function in the reverse direction. Alpha-keto acids such as pyruvate have been shown to play an important role in the growth of different AOA species (22, 23) as well as in other catalase-deficient species such as *Prochlorococcus* (24). Neither pyruvate nor its reaction product acetate were detected in relevant concentrations in cultures of *N. adriaticus*, *N. piranensis* and *N. maritimus* (data not shown), suggesting that pyruvate secretion is not a prevalent strategy to scavenge H_2_O_2_ in *Nitrosopumilus* cultures.

Surprisingly, universal stress proteins of the UspA family were more abundant in all three strains (although belonging to different OGs in each strain) when grown in co-culture with *Oceanicaulis alexandrii* or with catalase but were almost completely absent in cultures exposed to H_2_O_2_ (Fig. S2A-C), suggesting an alternative function of these enzymes in addition to their role in stress response. UspA has also been shown to be involved in cell growth retardation in stationary phase cells of *E. coli* (25). *Nitrosopumilus* cells that were already inhibited by H_2_O_2_ might not need additional growth suppressors and thus downregulate the expression of UspA. Additionally, the higher relative abundance of ribonucleotide reductase (main text) potentially indicates abnormal DNA/cell mass ratios (26) and ribonucleotide reductase activity has been shown to be regulated during oxidative stress (27). In contrast to Qin *et al.* (28), no changes in the relative abundance of proteins related to cobalamin (vitamin B12) biosynthesis were identified, which the authors suggested play a role in counterbalancing the accumulation of nitric oxide and the resulting depletion in the active cobalamin pool during unbalanced growth. Our results indicate that the mechanisms to cope with nutrient limitation (ammonia and copper in the study of Qin *et al.* (28)) are fundamentally different from the cellular response to H_2_O_2_, regardless of the unbalanced growth cells likely encounter during both stress conditions.

In addition to the inter-species response of *Nitrosopumilus* towards H_2_O_2_ exposure, some changes in the proteome composition were only shared by one or two strains, including signal transduction histidine kinases, as well as transcription factors and transcription regulation factors (Fig. S2A-C). In proteomes of *N. adriaticus* and *N. piranensis*, methionine synthase was detected at higher relative abundance. It has previously been suggested that methionine residues on the surface of proteins could function as efficient oxidant scavenger as a “last-chance” antioxidant defense system (29). Surface-exposed methionine residues that surround the entrance to the active site of enzymes are preferentially oxidized without loss of catalytic activity for proteins (28). Moreover, the reduction back to methionine by methionine sulfoxide reductases would allow the antioxidant system to function catalytically (29)*.*

Interestingly, the respiratory-chain enzyme F420H2 dehydrogenase subunit C was detected at lower relative abundance in *N. adriaticus* and *N. maritimus* when exposed to H_2_O_2_ (Fig. S2A,C). NADH:ubiquinone oxidoreductase (complex I) has been shown to be a major site of H_2_O_2_ production (30), hence downregulating one subunit of this complex could potentially influence the rate of H_2_O_2_ production. Furthermore, the DNA repair and recombination protein RadA was detected at higher relative abundance in *N. adriaticus* and *N. piranensis* when exposed to H_2_O_2_. RadA is homologous to bacterial RecA (31), which, in addition to its key role in DNA damage response has also been shown to affect responses to oxidative stress (32).

In *N. piranensis*, two heat-shock proteins, Hsp70 (DnaK) and Hsp20, were detected at higher relative abundance in the absence of H_2_O_2_ scavengers (Fig. S2B). Heat-shock proteins function as molecular chaperones which assist nascent proteins to reach their native fold, thereby minimizing their probability of aggregating into nonfunctional structures (33). Heat-shock proteins are typically induced upon protein-denaturing stress, indicating that *N. piranensis* cells were indeed stressed when exposed to H_2_O_2_ (33)_._ Additionally, one iron−sulfur cluster assembly accessory protein and protein−disulfide isomerase, proteins related to classical oxidative stress defense systems in bacteria (34), were present at higher relative abundance in *N. piranensis* (Fig. S2B). Interestingly, growth of *N. piranensis* was inhibited at lower H_2_O_2_ concentrations as compared to the other two strains (see main text). The greater sensitivity of *N. piranensis* coincides with the expression of proteins putatively involved in stress response, which were not part of the proteomic response in *N. adriaticus* and *N. maritimus*.

**Potential sources of H_2_O_2_ production in AOA**

The accidental autoxidation of flavin-containing proteins has been shown to be a major source of H_2_O_2_ (35, 36). In *E. coli*, predominant sources of cytoplasmic H_2_O_2_ are non-respiratory flavoproteins (37, 38) and to a lesser extent menaquinone autoxidation (via O_2_^-^ formation), however, enzymes responsible for approximately two third of the H_2_O_2_ produced by *E. coli* remain unknown (38). In AOA, saturated and monounsaturated menaquinones (MK_6:1_ and MK_6:0_) are the major respiratory quinones (39) and flavin-dependent oxidoreductases are part of the core AOA proteome (40, this study), suggesting an important role in their metabolic machinery. Furthermore, nitrite reductase in denitrifiers has been shown to produce H_2_O_2_ (41), which can be overcome by catalase addition (42). Interestingly, putative membrane-bound copper-containing nitrite reductases (NirK) are among the most highly expressed genes in some AOA (e.g., 40, 43, 44, this study), however, further studies are needed to identify sources of H_2_O_2_ in AOA.

**Supplementary References**

1. Bensadoun A, Weinstein D. 1976. Assay of proteins in the presence of interfering materials. *Anal Biochem* **70**:241–250.

2. Valledor L, Weckwerth W. 2014. An Improved Detergent-Compatible Gel-Fractionation LC-LTQ-Orbitrap-MS Workflow for Plant and Microbial Proteomics, p. 347–358. *In* Jorrin-Novo, J V, Komatsu, S, Weckwerth, W, Wienkoop, S (eds.), Plant Proteomics: Methods and Protocols. Humana Press, Totowa, NJ.

3. Shevchenko A, Tomas H, Havliš J, Olsen J V., Mann M. 2007. In-gel digestion for mass spectrometric characterization of proteins and proteomes. *Nat Protoc* **1**:2856–2860.

4. Smith CJ, Nedwell DB, Dong LF, Osborn AM. 2006. Evaluation of quantitative polymerase chain reaction-based approaches for determining gene copy and gene transcript numbers in environmental samples. *Environ Microbiol* **8**:804–815.

5. Wuchter C, Abbas B, Coolen MJL, Herfort L, Bleijswijk J Van, Timmers P, Strous M, Teira E, Herndl GJ, Middelburg JJ, Schouten S, Damste JSS. 2006. Archaeal nitrification in the ocean. *Proc Natl Acad Sci USA* **103**:12317–12322.

6. Marie D, Brussaard CPD, Thyrhaug R, Bratbak G, Vaulot D. 1999. Enumeration of marine viruses in culture and natural samples by flow cytometry. *Appl Environ Microbiol* **65**:45–52.

7. Teira E, Reinthaler T, Pernthaler A, Pernthaler J, Herndl GJ. 2004. Combining catalyzed reported deposition-fluorescence in situ hybridization and automicrography to detect substrate utilization by Bacteria and Archaea in the Deep Ocean. *Appl Environ Microbiol* **70**:4411–4414.

8. Woebken D, Burow LC, Prufert-Bebout L, Bebout BM, Hoehler TM, Pett-Ridge J, Spormann AM, Weber PK, Singer SW. 2012. Identification of a novel cyanobacterial group as active diazotrophs in a coastal microbial mat using NanoSIMS analysis. *ISME J* **6**:1427–1439.

9. Greuter D, Loy A, Horn M, Rattei T. 2016. probeBase –– an online resource for rRNA-targeted oligonucleotide probes and primers : new features 2016. *Nucleic Acids Res* **44**:D586–D589.

10. O’Leary NA, Wright MW, Brister JR, Ciufo S, Haddad D, McVeigh R, Rajput B, Robbertse B, Smith-White B, Ako-Adjei D, Astashyn A, Badretdin A, Bao Y, Blinkova O, Brover V, Chetvernin V, Choi J, Cox E, Ermolaeva O, Farrell CM, Goldfarb T, Gupta T, Haft D, Hatcher E, Hlavina W, Joardar VS, Kodali VK, Li W, Maglott D, Masterson P, McGarvey KM, Murphy MR, O’Neill K, Pujar S, Rangwala SH, Rausch D, Riddick LD, Schoch C, Shkeda A, Storz SS, Sun H, Thibaud-Nissen F, Tolstoy I, Tully RE, Vatsan AR, Wallin C, Webb D, Wu W, Landrum MJ, Kimchi A, Tatusova T, DiCuccio M, Kitts P, Murphy TD, Pruitt KD. 2016. Reference sequence (RefSeq) database at NCBI: current status, taxonomic expansion, and functional annotation. *Nucleic Acids Res* **44**:D733-745.

11. Passardi F, Theiler G, Zamocky M, Cosio C, Rouhier N, Teixera F, Margis-Pinheiro M, Ioannidis V, Penel C, Falquet L, Dunand C. 2007. PeroxiBase: The peroxidase database. *Phytochemistry* **68**:1605–1611.

12. Tamura K, Stecher G, Peterson D, Filipski A, Kumar S. 2013. MEGA6: Molecular Evolutionary Genetics Analysis version 6.0. *Mol Biol Evol* **30**:2725–9.

13. Zámocký M, Gasselhuber B, Furtmüller PG, Obinger C. 2014. Turning points in the evolution of peroxidase-catalase superfamily: Molecular phylogeny of hybrid heme peroxidases. *Cell Mol Life Sci* **71**:4681–4696.

14. Almagro Armenteros JJ, Tsirigos KD, Sønderby CK, Petersen TN, Winther O, Brunak S, von Heijne G, Nielsen H. 2019. SignalP 5.0 improves signal peptide predictions using deep neural networks. *Nat Biotechnol* doi:10.1038/s41587-019-0036-z.

15. Carlson CB, Bernstein DA, Annis DS, Misenheimer TM, Hannah BL, Mosher DF, Keck JL. 2005. Structure of the calcium-rich signature domain of human thrombospondin-2. *NatStructMolBiol* **12**:910–914.

16. Tan K, Duquette M, Joachimiak A, Lawler J. 2009. The crystal structure of the signature domain of cartilage oligomeric matrix protein: implications for collagen, glycosaminoglycan and integrin binding. *FASEB J* **23**:2490–2501.

17. Dai S, Sun C, Tan K, Ye S, Zhang R. 2017. Structure of thrombospondin type 3 repeats in bacterial outer membrane protein A reveals its intra-repeat disulfide bond-dependent calcium-binding capability. *Cell Calcium* **66**:78–89.

18. Adams JC. 2001. Thrombospondins: Multifunctional Regulators of Cell Interactions. *Annu Rev Cell Dev Biol* **17**:25–51.

19. Tan K, Lawler J. 2009. The interaction of Thrombospondins with extracellular matrix proteins. *J Cell Commun Signal* **3**:177–187.

20. Sylvestre P, Couture-Tosi E, Mock M. 2002. A collagen-like surface glycoprotein is a structural component of the Bacillus anthracis exosporium. *Mol Microbiol* **45**:169–178.

21. Hildebrand PW, Preissner R, Frömmel C. 2004. Structural features of transmembrane helices. *FEBS Lett* **559**:145–151.

22. Tourna M, Stieglmeier M, Spang A, Könneke M, Schintlmeister A, Urich T. 2011. Nitrososphaera viennensis, an ammonia oxidizing archaeon from soil. *Proc Natl Acad Sci USA* **108**:8420–8425.

23. Qin W, Amin SA, Martens-Habbena W, Walker CB, Urakawa H, Devol AH, Ingalls AE, Moffettt JW, Armbrust EV, Stahl DA. 2014. Marine ammonia-oxidizing archaeal isolates display obligate mixotrophy and wide ecotypic variation. *Proc Natl Acad Sci USA* **111**:12504–12509.

24. Ma L, Calfee BC, Morris JJ, Johnson ZI, Zinser ER. 2018. Degradation of hydrogen peroxide at the ocean’s surface: the influence of the microbial community on the realized thermal niche of Prochlorococcus. *ISME J* **12**:473–484.

25. Nyström T, Neidhardt FC. 1994. Expression and role of the universal stress protein, UspA, of Escherichia coii during growth arrest. *Mol Microbiol* **11**:537–544.

26. Herrick J, Sclavi B. 2007. Ribonucleotide reductase and the regulation of DNA replication: An old story and an ancient heritage. *Mol Microbiol* **63**:22–34.

27. Gon S, Beckwith J. 2006. Ribonucleotide Reductases: Influence of Environment on Synthesis and Activity. *Antioxidants Redox Signal* **8**:773–780.

28. Qin W, Amin SA, Lundeen RA, Heal KR, Martens-Habbena W, Turkarslan S, Urakawa H, Costa KC, Hendrickson EL, Wang T, Beck DA, Tiquia-Arashiro SM, Taub F, Holmes AD, Vajrala N, Berube PM, Lowe TM, Moffett JW, Devol AH, Baliga NS, Arp DJ, Sayavedra-Soto LA, Hackett M, Armbrust EV, Ingalls AE, Stahl DA. 2018. Stress response of a marine ammonia-oxidizing archaeon informs physiological status of environmental populations. *ISME J* **12**:508–519.

29. Levine RL, Mosoni L, Berlett BS, Stadtman ER. 1996. Methionine residues as endogenous antioxidants in proteins. *Proc Natl Acad Sci USA* **93**:15036–15040.

30. Esterházy D, King MS, Yakovlev G, Hirst J. 2008. Production of Reactive Oxygen Species by Complex I (NADH:Ubiquinone Oxidoreductase) from Escherichia coli and Comparison to the Enzyme from Mitochondria. *Biochemistry* **47**:3964–3971.

31. Seitz EM, Brockman JP, Sandler SJ, Clark AJ, Kowalczykowski SC. 1998. RadA protein is an archaeal RecA protein homolog that catalyzes DNA strand exchange. *Genes Dev* **12**:1248–1253.

32. Duwat P, Ehrlich SD, Gruss A. 1995. The recA gene of Lactococcus lactis: characterization and involvement in oxidative and thermal stress. *Mol Microbiol* **6**:1121–1131.

33. Lindquist S. 1992. Heat-shock proteins and stress tolerance in microorganisms. *Curr Opin Genet Dev* **2**:748–755.

34. Imlay JA. 2011. Cellular defenses against superoxide and hydrogen peroxide. *Annu Rev Biochem* **77**:755–776.

35. Messner KR, Imlay JA. 1999. The identification of primary sites of superoxide and hydrogen peroxide formation in the aerobic respiratory chain. *J Biol Chem* **274**:10119–28.

36. Massey V. 1994. Activation of Molecular Oxygen by Flavins and Flavoprotein. *J Biol Chem* **269**:22459–22462.

37. Seaver LC, Imlay JA. 2004. Are respiratory enzymes the primary sources of intracellular hydrogen peroxide? *J Biol Chem* **279**:48742–48750.

38. Korshunov S, Imlay J. 2011. Two sources of endogenous H2O2 in Escherichia coli. *Mol Microbiol* **75**:1389–1401.

39. Elling FJ, Becker KW, Könneke M, Schröder JM, Kellermann MY, Thomm M, Hinrichs K-U. 2016. Respiratory quinones in Archaea: phylogenetic distribution and application as biomarkers in the marine environment. *Environ Microbiol* **18**:692–707.

40. Kerou M, Offre P, Valledor L, Abby SS, Melcher M, Nagler M, Weckwerth W, Schleper C. 2016. Proteomics and comparative genomics of Nitrososphaera viennensis reveal the core genome and adaptations of archaeal ammonia oxidizers. *Proc Natl Acad Sci USA* **113**:E7937–E7946.

41. Kakutani T, Watanabe H, Arima K, Beppu T. 1981. A blue protein as an inactivating factor for nitrite reductase from Alcaligenes faecalis strain S-6. *J Biochem* **89**:463–472.

42. MacPherson IS, Rosell FI, Scofield M, Mauk AG, Murphy MEP. 2010. Directed evolution of copper nitrite reductase to a chromogenic reductant. *Protein Eng Des Sel* **23**:137–145.

43. Carini P, Dupont CL, Santoro AE. 2018. Patterns of thaumarchaeal gene expression in culture and diverse marine environments. *Environ Microbiol* doi: 10.1111/1462-2920.14107.

44. Hollibaugh JT, Gifford S, Sharma S, Bano N, Moran MA. 2011. Metatranscriptomic analysis of ammonia-oxidizing organisms in an estuarine bacterioplankton assemblage. *ISME J* **5**:866–878.
