# Supplementary material for: Proteomic Response of Three Marine Ammonia-Oxidizing Archaea to Hydrogen Peroxide and Their Metabolic Interactions with a Heterotrophic Alphaproteobacterium
Source: mSystems. 2019 Jun 25;4(4):e00181-19. doi: 10.1128/mSystems.00181-19 (PMC6593220; doi:10.1128/mSystems.00181-19)
Supplement: FIG S2 [file mSystems.00181-19-sf002.pdf]

A

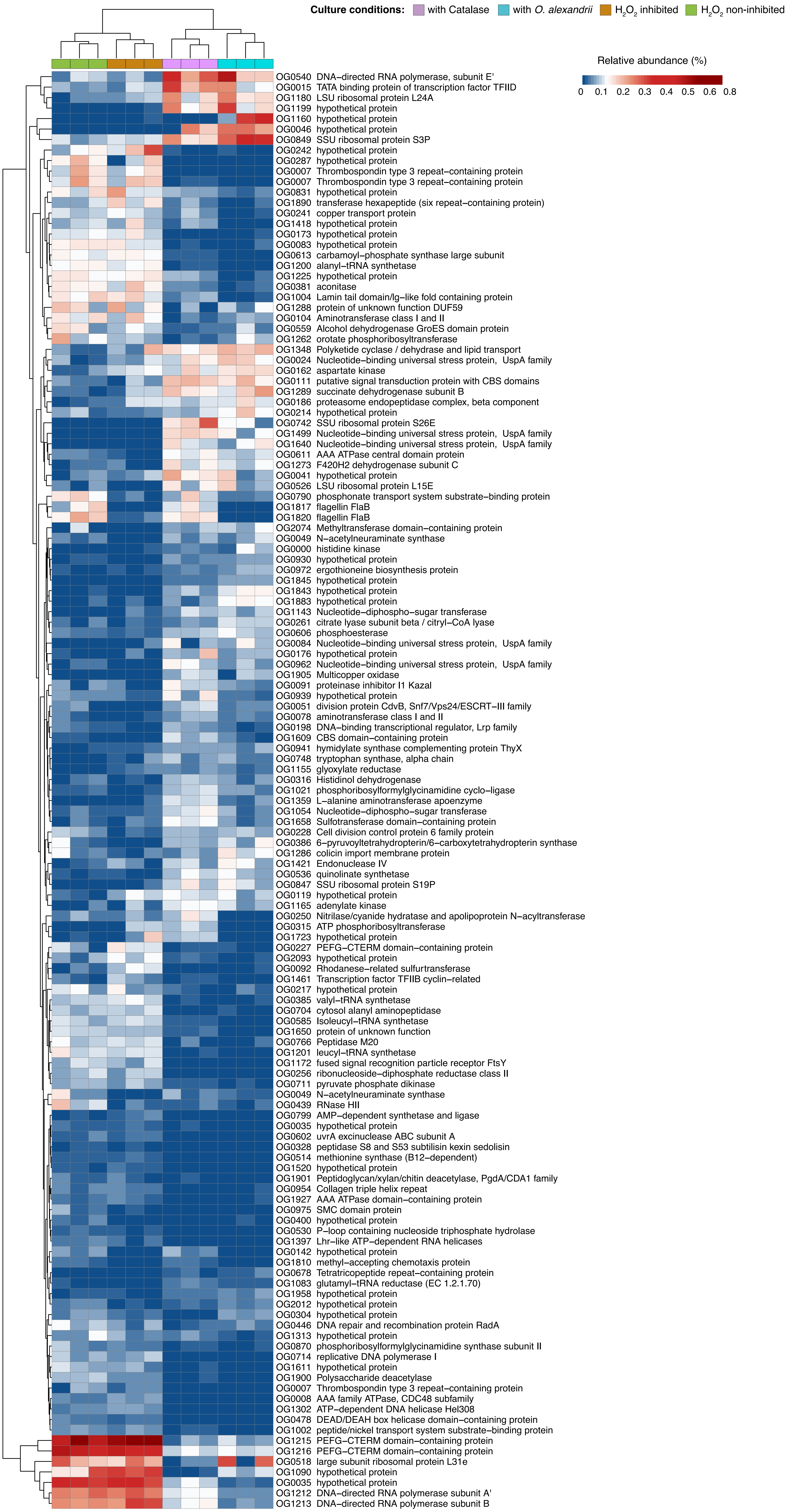

Culture conditions: with Catalase with *O. alexandrii* H_2O_2 inhibited H_2O_2 non-inhibited
Relative abundance (%)
0 0.1 0.2 0.3 0.4 0.5 0.6 0.8
OG0540 DNA–directed RNA polymerase, subunit E'
OG0015 TATA binding protein of transcription factor TFIID
OG1180 LSU ribosomal protein L24A
OG1199 hypothetical protein
OG1160 hypothetical protein
OG0046 hypothetical protein
OG0849 SSU ribosomal protein S3P
OG0242 hypothetical protein
OG0287 hypothetical protein
OG0007 Thrombospondin type 3 repeat–containing protein
OG0007 Thrombospondin type 3 repeat–containing protein
OG0831 hypothetical protein
OG1890 transferase hexapeptide (six repeat–containing protein)
OG0241 copper transport protein
OG1418 hypothetical protein
OG0173 hypothetical protein
OG0083 hypothetical protein
OG0613 carbamoyl–phosphate synthase large subunit
OG1200 alanyl–tRNA synthetase
OG1225 hypothetical protein
OG0381 aconitase
OG1004 Lamin tail domain/Ig–like fold containing protein
OG1288 protein of unknown function DUF59
OG0104 Aminotransferase class I and II
OG0559 Alcohol dehydrogenase GroES domain protein
OG1262 orotate phosphoribosyltransferase
OG1348 Polyketide cyclase / dehydrase and lipid transport
OG0024 Nucleotide–binding universal stress protein, UspA family
OG0162 aspartate kinase
OG0111 putative signal transduction protein with CBS domains
OG1289 succinate dehydrogenase subunit B
OG0186 proteasome endopeptidase complex, beta component
OG0214 hypothetical protein
OG0742 SSU ribosomal protein S26E
OG1499 Nucleotide–binding universal stress protein, UspA family
OG1640 Nucleotide–binding universal stress protein, UspA family
OG0611 AAA ATPase central domain protein
OG1273 F420H2 dehydrogenase subunit C
OG0041 hypothetical protein
OG0526 LSU ribosomal protein L15E
OG0790 phosphonate transport system substrate–binding protein
OG1817 flagellin FlaB
OG1820 flagellin FlaB
OG2074 Methyltransferase domain–containing protein
OG0049 N–acetylneuraminate synthase
OG0000 histidine kinase
OG0930 hypothetical protein
OG0972 ergothioneine biosynthesis protein
OG1845 hypothetical protein
OG1843 hypothetical protein
OG1883 hypothetical protein
OG1143 Nucleotide–diphospho–sugar transferase
OG0261 citrate lyase subunit beta / citryl–CoA lyase
OG0606 phosphoesterase
OG0084 Nucleotide–binding universal stress protein, UspA family
OG0176 hypothetical protein
OG0962 Nucleotide–binding universal stress protein, UspA family
OG1905 Multicopper oxidase
OG0091 proteinase inhibitor I1 Kazal
OG0939 hypothetical protein
OG0051 division protein CdvB, Snf7/Vps24/ESCRT–III family
OG0078 aminotransferase class I and II
OG0198 DNA–binding transcriptional regulator, Lrp family
OG1609 CBS domain–containing protein
OG0941 hymidylate synthase complementing protein ThyX
OG0748 tryptophan synthase, alpha chain
OG1155 glyoxylate reductase
OG0316 Histidinol dehydrogenase
OG1021 phosphoribosylformylglycinamidine cyclo–ligase
OG1359 L–alanine aminotransferase apoenzyme
OG1054 Nucleotide–diphospho–sugar transferase
OG1658 Sulfotransferase domain–containing protein
OG0228 Cell division control protein 6 family protein
OG0386 6–pyruvoyltetrahydropterin/6–carboxytetrahydropterin synthase
OG1286 colicin import membrane protein
OG1421 Endonuclease IV
OG0536 quinolinate synthetase
OG0847 SSU ribosomal protein S19P
OG0119 hypothetical protein
OG1165 adenylate kinase
OG0250 Nitrilase/cyanide hydratase and apolipoprotein N–acyltransferase
OG0315 ATP phosphoribosyltransferase
OG1723 hypothetical protein
OG0227 PEFG–CTERM domain–containing protein
OG2093 hypothetical protein
OG0092 Rhodanese–related sulfurtransferase
OG1461 Transcription factor TFIIB cyclin–related
OG0217 hypothetical protein
OG0385 valyl–tRNA synthetase
OG0704 cytosol alanyl aminopeptidase
OG0585 Isoleucyl–tRNA synthetase
OG1650 protein of unknown function
OG0766 Peptidase M20
OG1201 leucyl–tRNA synthetase
OG1172 fused signal recognition particle receptor FtsY
OG0256 ribonucleoside–diphosphate reductase class II
OG0711 pyruvate phosphate dikinase
OG0049 N–acetylneuraminate synthase
OG0439 RNase HII
OG0799 AMP–dependent synthetase and ligase
OG0035 hypothetical protein
OG0602 uvrA excinuclease ABC subunit A
OG0328 peptidase S8 and S53 subtilisin kexin sedolisin
OG0514 methionine synthase (B12–dependent)
OG1520 hypothetical protein
OG1901 Peptidoglycan/xylan/chitin deacetylase, PgdA/CDA1 family
OG0954 Collagen triple helix repeat
OG1927 AAA ATPase domain–containing protein
OG0975 SMC domain protein
OG0400 hypothetical protein
OG0530 P–loop containing nucleoside triphosphate hydrolase
OG1397 Lhr–like ATP–dependent RNA helicases
OG0142 hypothetical protein
OG1810 methyl–accepting chemotaxis protein
OG0678 Tetratricopeptide repeat–containing protein
OG1083 glutamyl–tRNA reductase (EC 1.2.1.70)
OG1958 hypothetical protein
OG2012 hypothetical protein
OG0304 hypothetical protein
OG0446 DNA repair and recombination protein RadA
OG1313 hypothetical protein
OG0870 phosphoribosylformylglycinamidine synthase subunit II
OG0714 replicative DNA polymerase I
OG1611 hypothetical protein
OG1900 Polysaccharide deacetylase
OG0007 Thrombospondin type 3 repeat–containing protein
OG0008 AAA family ATPase, CDC48 subfamily
OG1302 ATP–dependent DNA helicase Hel308
OG0478 DEAD/DEAH box helicase domain–containing protein
OG1002 peptide/nickel transport system substrate–binding protein
OG1215 PEFG–CTERM domain–containing protein
OG1216 PEFG–CTERM domain–containing protein
OG0518 large subunit ribosomal protein L31e
OG1090 hypothetical protein
OG0035 hypothetical protein
OG1212 DNA–directed RNA polymerase subunit A'
OG1213 DNA–directed RNA polymerase subunit B

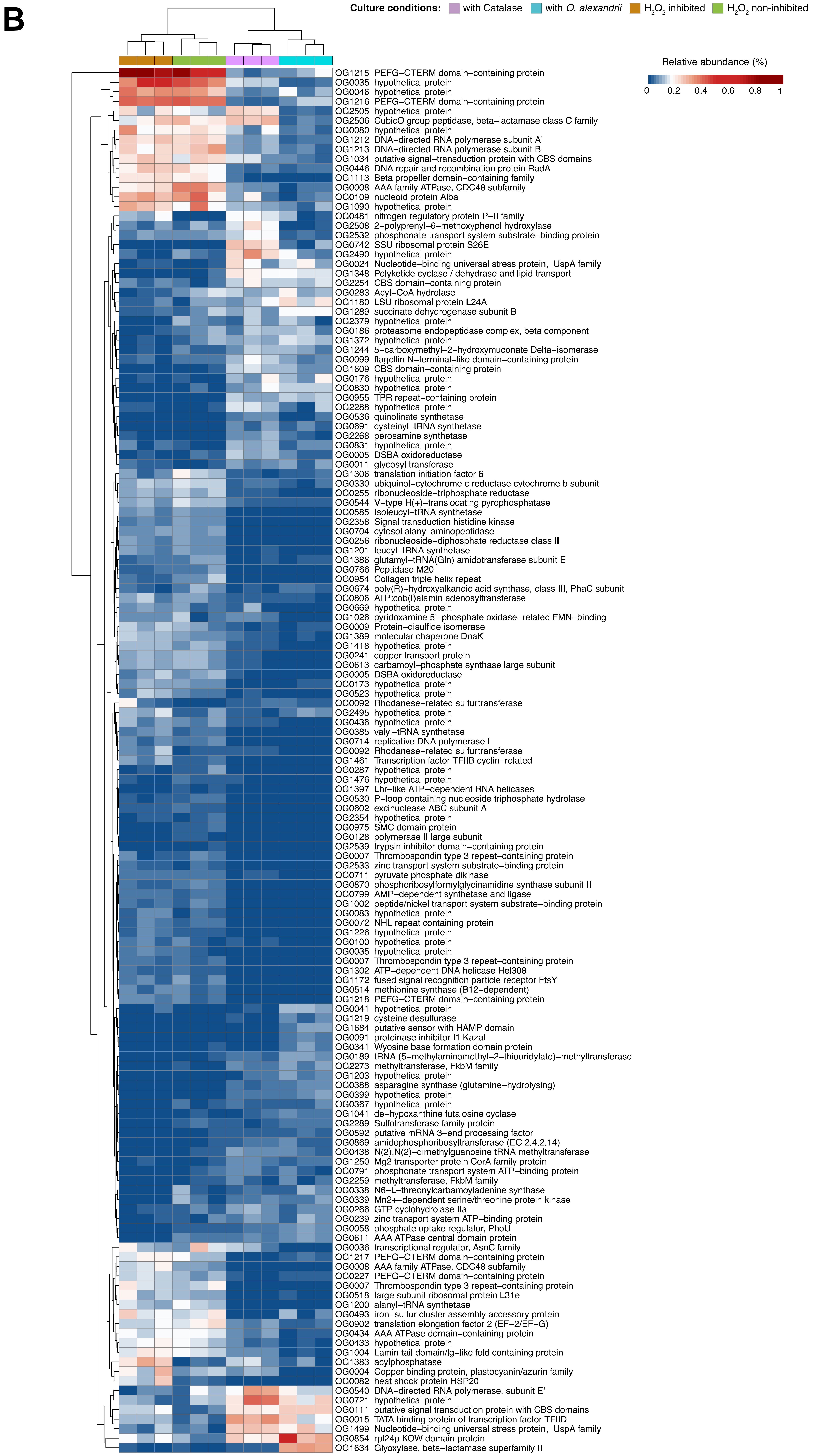
B
Culture conditions: with Catalase with O. alexandrii H2O2 inhibited H2O2 non-inhibited
Relative abundance (%)
0 0.2 0.4 0.6 0.8 1
OG1215 PEFG–CTERM domain–containing protein
OG0035 hypothetical protein
OG0046 hypothetical protein
OG1216 PEFG–CTERM domain–containing protein
OG2505 hypothetical protein
OG2506 CubicO group peptidase, beta–lactamase class C family
OG0080 hypothetical protein
OG1212 DNA–directed RNA polymerase subunit A'
OG1213 DNA–directed RNA polymerase subunit B
OG1034 putative signal–transduction protein with CBS domains
OG0446 DNA repair and recombination protein RadA
OG1113 Beta propeller domain–containing family
OG0008 AAA family ATPase, CDC48 subfamily
OG0109 nucleoid protein Alba
OG1090 hypothetical protein
OG0481 nitrogen regulatory protein P–II family
OG2508 2–polyprenyl–6–methoxyphenol hydroxylase
OG2532 phosphonate transport system substrate–binding protein
OG0742 SSU ribosomal protein S26E
OG2490 hypothetical protein
OG0024 Nucleotide–binding universal stress protein, UspA family
OG1348 Polyketide cyclase / dehydrase and lipid transport
OG2254 CBS domain–containing protein
OG0283 Acyl–CoA hydrolase
OG1180 LSU ribosomal protein L24A
OG1289 succinate dehydrogenase subunit B
OG2379 hypothetical protein
OG0186 proteasome endopeptidase complex, beta component
OG1372 hypothetical protein
OG1244 5–carboxymethyl–2–hydroxymuconate Delta–isomerase
OG0099 flagellin N–terminal–like domain–containing protein
OG1609 CBS domain–containing protein
OG0176 hypothetical protein
OG0830 hypothetical protein
OG0955 TPR repeat–containing protein
OG2288 hypothetical protein
OG0536 quinolinate synthetase
OG0691 cysteinyl–tRNA synthetase
OG2268 perosamine synthetase
OG0831 hypothetical protein
OG0005 DSBA oxidoreductase
OG0011 glycosyl transferase
OG1306 translation initiation factor 6
OG0330 ubiquinol–cytochrome c reductase cytochrome b subunit
OG0255 ribonucleoside–triphosphate reductase
OG0544 V–type H(+)–translocating pyrophosphatase
OG0585 Isoleucyl–tRNA synthetase
OG2358 Signal transduction histidine kinase
OG0704 cytosol alanyl aminopeptidase
OG0256 ribonucleoside–diphosphate reductase class II
OG1201 leucyl–tRNA synthetase
OG1386 glutamyl–tRNA(Gln) amidotransferase subunit E
OG0766 Peptidase M20
OG0954 Collagen triple helix repeat
OG0674 poly(R)–hydroxyalkanoic acid synthase, class III, PhaC subunit
OG0806 ATP:cob(I)alamin adenosyltransferase
OG0669 hypothetical protein
OG1026 pyridoxamine 5'–phosphate oxidase–related FMN–binding
OG0009 Protein–disulfide isomerase
OG1389 molecular chaperone DnaK
OG1418 hypothetical protein
OG0241 copper transport protein
OG0613 carbamoyl–phosphate synthase large subunit
OG0005 DSBA oxidoreductase
OG0173 hypothetical protein
OG0523 hypothetical protein
OG0092 Rhodanese–related sulfurtransferase
OG2495 hypothetical protein
OG0436 hypothetical protein
OG0385 valyl–tRNA synthetase
OG0714 replicative DNA polymerase I
OG0092 Rhodanese–related sulfurtransferase
OG1461 Transcription factor TFIIB cyclin–related
OG0287 hypothetical protein
OG1476 hypothetical protein
OG1397 Lhr–like ATP–dependent RNA helicases
OG0530 P–loop containing nucleoside triphosphate hydrolase
OG0602 excinuclease ABC subunit A
OG2354 hypothetical protein
OG0975 SMC domain protein
OG0128 polymerase II large subunit
OG2539 trypsin inhibitor domain–containing protein
OG0007 Thrombospondin type 3 repeat–containing protein
OG2533 zinc transport system substrate–binding protein
OG0711 pyruvate phosphate dikinase
OG0870 phosphoribosylformylglycinamidine synthase subunit II
OG0799 AMP–dependent synthetase and ligase
OG1002 peptide/nickel transport system substrate–binding protein
OG0083 hypothetical protein
OG0072 NHL repeat containing protein
OG1226 hypothetical protein
OG0100 hypothetical protein
OG0035 hypothetical protein
OG0007 Thrombospondin type 3 repeat–containing protein
OG1302 ATP–dependent DNA helicase Hel308
OG1172 fused signal recognition particle receptor FtsY
OG0514 methionine synthase (B12–dependent)
OG1218 PEFG–CTERM domain–containing protein
OG0041 hypothetical protein
OG1219 cysteine desulfurase
OG1684 putative sensor with HAMP domain
OG0091 proteinase inhibitor I1 Kazal
OG0341 Wyosine base formation domain protein
OG0189 tRNA (5–methylaminomethyl–2–thiouridylate)–methyltransferase
OG2273 methyltransferase, FkbM family
OG1203 hypothetical protein
OG0388 asparagine synthase (glutamine–hydrolysing)
OG0399 hypothetical protein
OG0367 hypothetical protein
OG1041 de–hypoxanthine futalosine cyclase
OG2289 Sulfotransferase family protein
OG0592 putative mRNA 3–end processing factor
OG0869 amidophosphoribosyltransferase (EC 2.4.2.14)
OG0438 N(2),N(2)–dimethylguanosine tRNA methyltransferase
OG1250 Mg2 transporter protein CorA family protein
OG0791 phosphonate transport system ATP–binding protein
OG2259 methyltransferase, FkbM family
OG0338 N6–L–threonylcarbamoyladenine synthase
OG0339 Mn2+–dependent serine/threonine protein kinase
OG0266 GTP cyclohydrolase IIa
OG0239 zinc transport system ATP–binding protein
OG0058 phosphate uptake regulator, PhoU
OG0611 AAA ATPase central domain protein
OG0036 transcriptional regulator, AsnC family
OG1217 PEFG–CTERM domain–containing protein
OG0008 AAA family ATPase, CDC48 subfamily
OG0227 PEFG–CTERM domain–containing protein
OG0007 Thrombospondin type 3 repeat–containing protein
OG0518 large subunit ribosomal protein L31e
OG1200 alanyl–tRNA synthetase
OG0493 iron–sulfur cluster assembly accessory protein
OG0902 translation elongation factor 2 (EF–2/EF–G)
OG0434 AAA ATPase domain–containing protein
OG0433 hypothetical protein
OG1004 Lamin tail domain/Ig–like fold containing protein
OG1383 acylphosphatase
OG0004 Copper binding protein, plastocyanin/azurin family
OG0082 heat shock protein HSP20
OG0540 DNA–directed RNA polymerase, subunit E'
OG0721 hypothetical protein
OG0111 putative signal transduction protein with CBS domains
OG0015 TATA binding protein of transcription factor TFIID
OG1499 Nucleotide–binding universal stress protein, UspA family
OG0854 rpl24p KOW domain protein
OG1634 Glyoxylase, beta–lactamase superfamily II

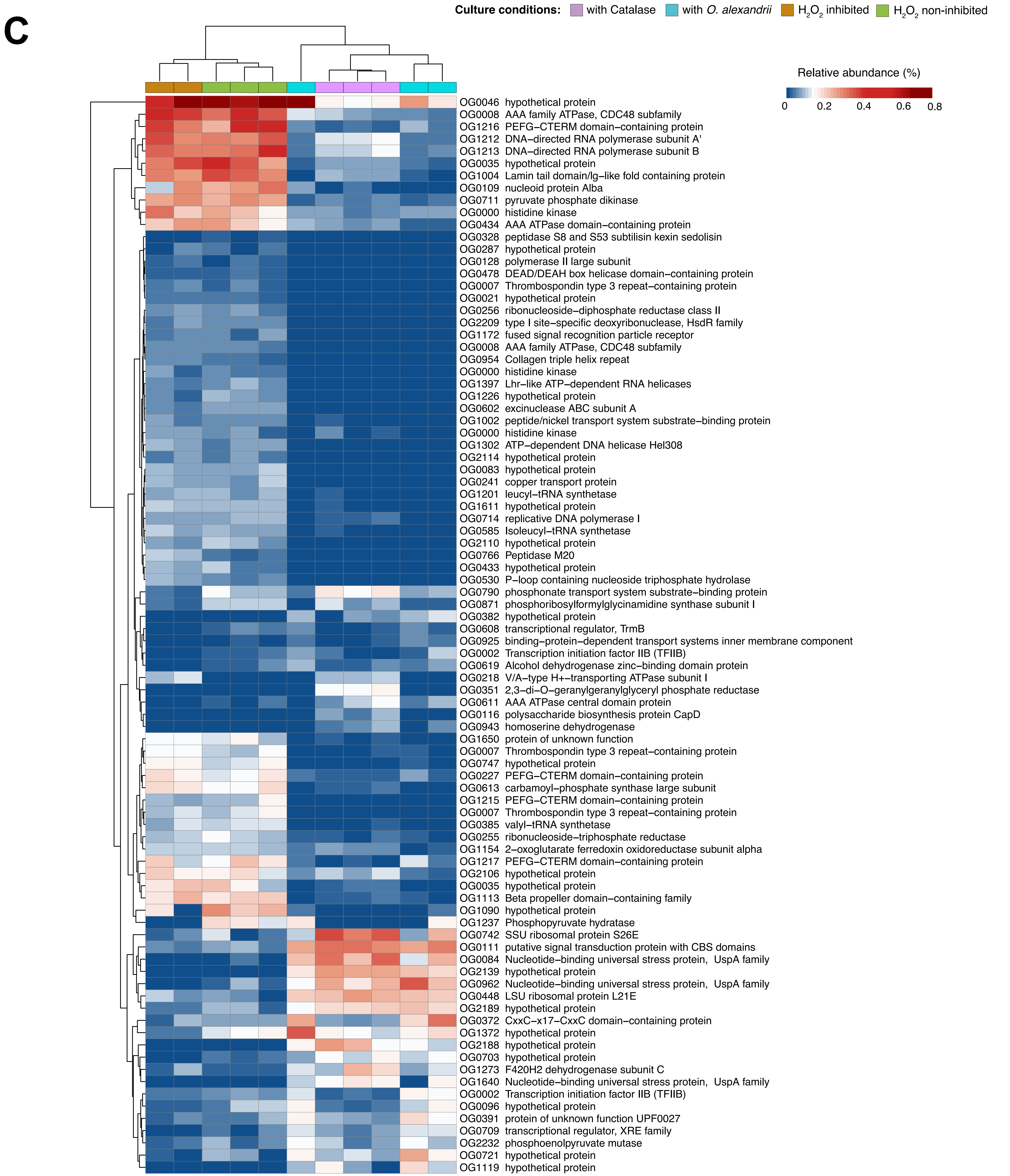
C
Culture conditions: with Catalase with O. alexandrii H2O2 inhibited H2O2 non-inhibited
Relative abundance (%)
0 0.2 0.4 0.6 0.8
OG0046 hypothetical protein
OG0008 AAA family ATPase, CDC48 subfamily
OG1216 PEFG–CTERM domain–containing protein
OG1212 DNA–directed RNA polymerase subunit A'
OG1213 DNA–directed RNA polymerase subunit B
OG0035 hypothetical protein
OG1004 Lamin tail domain/Ig–like fold containing protein
OG0109 nucleoid protein Alba
OG0711 pyruvate phosphate dikinase
OG0000 histidine kinase
OG0434 AAA ATPase domain–containing protein
OG0328 peptidase S8 and S53 subtilisin kexin sedolisin
OG0287 hypothetical protein
OG0128 polymerase II large subunit
OG0478 DEAD/DEAH box helicase domain–containing protein
OG0007 Thrombospondin type 3 repeat–containing protein
OG0021 hypothetical protein
OG0256 ribonucleoside–diphosphate reductase class II
OG2209 type I site–specific deoxyribonuclease, HsdR family
OG1172 fused signal recognition particle receptor
OG0008 AAA family ATPase, CDC48 subfamily
OG0954 Collagen triple helix repeat
OG0000 histidine kinase
OG1397 Lhr–like ATP–dependent RNA helicases
OG1226 hypothetical protein
OG0602 excinuclease ABC subunit A
OG1002 peptide/nickel transport system substrate–binding protein
OG0000 histidine kinase
OG1302 ATP–dependent DNA helicase Hel308
OG2114 hypothetical protein
OG0083 hypothetical protein
OG0241 copper transport protein
OG1201 leucyl–tRNA synthetase
OG1611 hypothetical protein
OG0714 replicative DNA polymerase I
OG0585 Isoleucyl–tRNA synthetase
OG2110 hypothetical protein
OG0766 Peptidase M20
OG0433 hypothetical protein
OG0530 P–loop containing nucleoside triphosphate hydrolase
OG0790 phosphonate transport system substrate–binding protein
OG0871 phosphoribosylformylglycinamidine synthase subunit I
OG0382 hypothetical protein
OG0608 transcriptional regulator, TrmB
OG0925 binding–protein–dependent transport systems inner membrane component
OG0002 Transcription initiation factor IIB (TFIIB)
OG0619 Alcohol dehydrogenase zinc–binding domain protein
OG0218 V/A–type H+–transporting ATPase subunit I
OG0351 2,3–di–O–geranylgeranylglyceryl phosphate reductase
OG0611 AAA ATPase central domain protein
OG0116 polysaccharide biosynthesis protein CapD
OG0943 homoserine dehydrogenase
OG1650 protein of unknown function
OG0007 Thrombospondin type 3 repeat–containing protein
OG0747 hypothetical protein
OG0227 PEFG–CTERM domain–containing protein
OG0613 carbamoyl–phosphate synthase large subunit
OG1215 PEFG–CTERM domain–containing protein
OG0007 Thrombospondin type 3 repeat–containing protein
OG0385 valyl–tRNA synthetase
OG0255 ribonucleoside–triphosphate reductase
OG1154 2–oxoglutarate ferredoxin oxidoreductase subunit alpha
OG1217 PEFG–CTERM domain–containing protein
OG2106 hypothetical protein
OG0035 hypothetical protein
OG1113 Beta propeller domain–containing family
OG1090 hypothetical protein
OG1237 Phosphopyruvate hydratase
OG0742 SSU ribosomal protein S26E
OG0111 putative signal transduction protein with CBS domains
OG0084 Nucleotide–binding universal stress protein, UspA family
OG2139 hypothetical protein
OG0962 Nucleotide–binding universal stress protein, UspA family
OG0448 LSU ribosomal protein L21E
OG2189 hypothetical protein
OG0372 CxxC–x17–CxxC domain–containing protein
OG1372 hypothetical protein
OG2188 hypothetical protein
OG0703 hypothetical protein
OG1273 F420H2 dehydrogenase subunit C
OG1640 Nucleotide–binding universal stress protein, UspA family
OG0002 Transcription initiation factor IIB (TFIIB)
OG0096 hypothetical protein
OG0391 protein of unknown function UPF0027
OG0709 transcriptional regulator, XRE family
OG2232 phosphoenolpyruvate mutase
OG0721 hypothetical protein
OG1119 hypothetical protein
