# Supplementary material for: Proteomic Response of Three Marine Ammonia-Oxidizing Archaea to Hydrogen Peroxide and Their Metabolic Interactions with a Heterotrophic Alphaproteobacterium
Source: mSystems. 2019 Jun 25;4(4):e00181-19. doi: 10.1128/mSystems.00181-19 (PMC6593220; doi:10.1128/mSystems.00181-19)
Supplement: DATA SET S2 [file mSystems.00181-19-sd002.docx]

**(A)**

**AB-661-L21:**

>H944DRAFT_00361

MRIAKIVGDVTLSRCLPEYVGASLRVAVALTLDELPRNGAQAETDNGEILVLWDEFASGH

GSLIALSDGAEAARPFRPVNRPVDAYNAAILENIIVK

>H944DRAFT_00362

MNNVFKIKQDICEIGRRIYQKEFAAANDGNISVRIGDNEVLCTPTMQSKGFLTPDDICTI

DMTGKQTAGRKKRSSEALLHLEIYKARADIKSVVHCHPPHATAFAIAHEPIPQCVLPEVE

IFLGDVPITRYETPGGKEFAETILPFVDKSNVIILANHGTVSFGEDVEKAYWWTEILDAY

CRMLMLARGLGNISYFSEEKQRELLALKDEWGYSDPRNTKEFEDCDICANDVFRDSWSES

GVQRRSFSPPPAMGGGANADANSMDQDDLVREITERVMAALSQSGNVA

>H944DRAFT_00363

MKVSIIGAGGLVGSSAAFALQCGGVVREMALLDVNLEAAEGQALDIMHGGPSTADQIIVA

GGYEHVVDSDVICITAGLRRQPDESRLDLINRNTDLFVGILDEINTVGTKPGAIVFVVSN

PVDILTYVAAQRLGLPLSQVIGLGTQLDTIRFRSLIAAELGIAPTQTSALILGEHGESMV

PIWSSATFAGVALDRHPNWTPALASELFTRTRGSGSEVIRKKGGAGFAVGIAIRDVIESI

ALDSQKVLPVSSVQEGCYGIQDVALSVPTVIGRAGVIDRHEIDLWPKEIQGLRASGNALR

KTIATVMQRIGS

>H944DRAFT_00364

MDLQYICSCYTHYFKPSNANLESDPMNRAVLTSLLLFVILLPLTATAQRNHRLDAFKHIE

VLADDSFEGRRAGSTGGHAAAGYIVKHLAEFGLTPAGEESYFQPFNKKYENCRNILAILP

GKHPKLNEEIVAIGAHYDHVGYGQGKNRGPVHNGADDNASGTATVLELARYFSKPRNRPE

RSLMFCFWDSEEQGLIGSKYWVEQPTVELSKIVFKVNIDMIGRLEGETLEVQGHRSGNGI

KEPLNAANAKLGIDIDYVFNMPANSDHWPFINKGIPGFMFHTGLHPEYHKPGDDSETIDV

EGLEQVGQLARNFITFLATTKSPPTFRKESLSEKPPAK

>H944DRAFT_00366

VFEKVTRRFVVIVMALGAFLGLGRKRSPAASIDDALERIEQWDRTHDRVWLGGEFWSNPM

EDWRIQDGWVQCQTTAANRSIHSVTRQLTDFGSPFAMSVELSQSVAIEKDGGAGFRIGIR

SDLNEYRSNCFAKNGINAGLLGKNLILGLSRAPLAGELNNSHVLLTLQGRPQGDNCVLTL

TARDSGSGKEVGRIQQSFPQESIRGNIALVSQYLGVQRRVPSPGYRFRNWSIGGAAITHE

PKQTFGPVLWTMYSLSDSRGKEGFVLKLTAITGPLGKDDNQKVELQIKRRGKWMTLGKAK

LDREAWTATFRIANWNETQATPYRVIYEQSFKEGDSITDRWHGTIRANPVGRPLRLAALT

CQNDYAFPYAPVADNVVKMNPDMIYFSGDQLYEGHGGFGIIRDPADPAILNYLRKFYQFG

WSFRESMKDAPTICIPDDHDVFQGNIWGEAGAAMKDLDKGASSKGGYREPARMVNVVHMT

NCSHHPDFYDPQPVEQDISVYFGDMVYGGVSFAILGDRQWKSGPERVETGSGRADHVRDK

DFDTAVLDKPDLVLLGDRQEAFLKAWGKDWRGHTMKVLLSQTVFAGVATHHGNFNGYLKA

DLDSGAWPQTARNRAIDIIRESMALHINGDQHLASLCQYGVNEQRDSNWSFCTPAIGAGY

PRWWRPDEVGMPHQNRPQHGLAHTGEYLDGFGNKVYVYCTGNPEVATKRGRYERAHQKGS

GFGFVTIDTLKKTYTLDAYQFNADPTDGNPDNQFPGWPITLAQAENKGQNRDH

>H944DRAFT_00367

MGDDFSYADEFKTVDLAALQADIFELMTTSQDWWPADYGHYGPFFIRMAWHSAGTYRIAD

GRGGAASGTLRFAPLNSWPDNVNLDKARRLLWPIKQKYGKTLSWADLMVFTGNCALESMG

LKTFGFAGGREDVWEPEIDISWGAESEWLGDERYSGDRDLANPLGAVQMGLIYVNPEGPN

GTPDPLASAVDIRETFRRMAMNDEETVALIAGGHTFGKAHGAADPEQYVGPEPEGANLDD

QGLGWKNSFGTGNAGDTISSGLEGAWTTEPAKWDNNYFDNLFGYEWEQVKSPAGATQWTP

TDVSAQATVADAHDPDKKHAPMMFTTDMALKMDPVYGPISKRFHENPEEFADAFAKAWYK

LTHRDMGPVSRCLGPLVPEPQLWQDPIPETTHETISDEDVTELKTQILASDISLSQLVST

AWASATTFRATDNRGGANGARLRLAPQKDWDANVPAQLAGVLQVLEGIQSTFNSSQADGK

QVSLADLIVLGGTAAIEEAAKQAGTDVAVPFTAGRADASQEQTDVESFAVLEPAADGFRN

YAGADLGFPAEELLIDRAHLLTLTAPEMTVLVGGLRVLNVTAGDSGLGVFTKNPETLTND

FFVNLLDNSIKWQKSSKCEHFYEGCDRDSGEIKWMGTAVDLVFGSNSQLRAVAEAYACDD

SQQAFVVDFVAAWNKVMNLDRFDV

>H944DRAFT_00368

MFVYLLCFTSLVAQDKTGQNKATPVSHIKVVPGFEVELLYSVPSKDQGSWVNLCVDNKGR

IIVSDQFGGLYRMQPPAAGSPLDPASIETVPAPIRAVNGMVWAFDALYVGVNDYEQKISS

GLYRLTDSTGDDQLDKVELLREVKSRGDHGVHAVVPTPDGKSLFLITGNNSKPPIIEPTS

PVTQVWGEDHLLPSMPDGRGHNRGVLAPGGIIYKVSPDGKQFEAYASGFRNIFGGAINRD

GELFTYDADMEYDFNTPWYRPTRICLVTSGAEFGWRNGAGKRPVFYPDNLPGVLDIGPGS

PTGMTFGYGAKFPAKYQNALYSLDWSWGKLYAVHLKPDGAGYTATKEEFVTGAPLPITDA

MIHPGDGAMYFTIGGRRVQSGLYRVTYVGNESTRAVSTKPTVTAERQTRHMLEEFHGEPN

PKALNAAWPLLDHQDRLIRWAARMAVEHQPTEQWTERALQDPNPNTQVEALLALCRMTGI

CPQHRTDQSPPVDEAMGQKLIQALLAVNTAKLKPAQQLTLVRTLQITLIRFGRPEHAIVT

ELNQKFDPLFPADTFEENWLLCETLAWLESPTVAAKTLALIQNAATQEEQLQYARSLRML

QAGWNTELRTQYFNWFLKADSYRGGASFDIFLKFIRDDAVASLDAKSKTSLAQLLATKPV

RKSVIENLGAIFEGRETQDWSLDTLSAAANADLKDRNYDNGKRMFAAAACYACHRFNNQG

GITGPDLTSAGRRYSPHDLLDQIINPSKVINDQFSAVVVITDAGVIHTGVVVNLNGDSLM

LNTDLTNPNQQVRLNRNEIDEINISKVSPMPKGLLAKMTREEILDLVAYVISAGDSKHKV

FKE

>H944DRAFT_00369

DSTSVALIALNRNQNSRAIEVRDANELRKDAYPQILESLLNDTETDGSVGVLNAGILSYS

PLLEEQMLEHVVKHYRPHVVTLMLDCTDIGDDYHYSLEYDPQQLGGPFSGPDVTKPKPHF

GALWRFAKPLHPTLLAPFKLLDRLGGGYQPLDPFDYYKFRLLVAGKVETERFFIYRHPLD

VTRQYFDATWDTINRIAATCRQQDAEFILYIAPRYHHWNAKESPDNWETFAYGLEEPYQF

EIFNYFESKIDSAPFKIVNMLPTFQATQEYPLVFRTDPHWNPQGNRFVAHLLQRSLTNSQ

SK

**AB-661-M19:**

>H950DRAFT_00391

DSTSVALIALNRNQNSRAIEVRDANELRKDANTTCANIFVVCKNSQSARLIKQIKLVPQH

TGSPPEWL

>H950DRAFT_00392

MRIAKIVGDVTLSRCLPEYVGASLRVAVALTLDELPRNGAQAETDNGEILVLWDEFASGH

GSLIALSDGAEAARPFRPVNRPVDAYNAAILENIIVK

>H950DRAFT_00393

MNNVFKIKQDICEIGRRIYQKEFAAANDGNISVRIGDNEVLCTPTMQSKGFLTPDDICTI

DMTGKQTAGRKKRSSEALLHLEIYKARADIKSVVHCHPPHATAFAIAHEPIPQCVLPEVE

IFLGDVPITRYETPGGKEFAETILPFVDKSNVIILANHGTVSFGEDVEKAYWWTEILDAY

CRMLMLARGLGNISYFSEEKQRELLALKDEWGYSDPRNTKEFEDCDICANDVFRDSWSES

GVQRRSFSPPPAMGGGANADANSMDQDDLVREITERVMAALSQSGNVA

>H950DRAFT_00394

MKVSIIGAGGLVGSSAAFALQCGGVVREMALLDVNLEAAEGQALDIMHGGPSTADQIIVA

GGYEHVVDSDVICITAGLRRQPDESRLDLINRNTDLFVGILDEINTVGTKPGAIVFVVSN

PVDILTYVAAQRLGLPLSQVIGLGTQLDTIRFRSLIAAELGIAPTQTSALILGEHGESMV

PIWSSATFAGVALDRHPNWTPALASELFTRTRGSGSEVIRKKGGAGFAVGIAIRDVIESI

ALDSQKVLPVSSVQEGCYGIQDVALSVPTVIGRAGVIDRHEIDLWPKEIQGLRASGNALR

KTIATVMQRIGS

>H950DRAFT_00395

MNRAVLTSLLLFVILLPLTATAQRNHRLDAFKHIEVLADDSFEGRRAGSTGGHAAAGYIV

KHLAEFGLTPAGEESYFQPFNKKYENCRNILAILPGKHPKLNEEIVAIGAHYDHVGYGQG

KNRGPVHNGADDNASGTATVLELARYFSKPRNRPERSLMFCFWDSEEQGLIGSKYWVEQP

TVELSKIVFKVNIDMIGRLEGETLEVQGHRSGNGIKEPLNAANAKLGIDIDYVFNMPANS

DHWPFINKGIPGFMFHTGLHPEYHKPGDDSETIDVEGLEQVGQLARNFITFLATTKSPPT

FRKESLSEKPPAK

>H950DRAFT_00397

VFEKVTRRFVVIVMALGAFLGLGRKRSPAASIDDALERIEQWDRTHDRVWLGGEFWSNPM

EDWRIQDGWVQCQTTAANRSIHSVTRQLTDFGSPFAMSVELSQSVAIEKDGGAGFRIGIR

SDLNEYRSNCFAKNGINAGLLGKNLILGLSRAPLAGELNNSHVLLTLQGRPQGDNCVLTL

TARDSGSGKEVGRIQQSFPQESIRGNIALVSQYLGVQRRVPSPGYRFRNWSIGGAAITHE

PKQTFGPVLWTMYSLSDSRGKEGFVLKLTAITGPLGKDDNQKVELQIKRRGKWMTLGKAK

LDREAWTATFRIANWNETQATPYRVIYEQSFKEGDSITDRWHGTIRANPVGRPLRLAALT

CQNDYAFPYAPVADNVVKMNPDMIYFSGDQLYEGHGGFGIIRDPADPAILNYLRKFYQFG

WSFRESMKDAPTICIPDDHDVFQGNIWGEAGAAMKDLDKGASSKGGYREPARMVNVVHMT

NCSHHPDFYDPQPVEQDISVYFGDMVYGGVSFAILGDRQWKSGPERVETGSGRADHVRDK

DFDTAVLDKPDLVLLGDRQEAFLKAWGKDWRGHTMKVLLSQTVFAGVATHHGNFNGYLKA

DLDSGAWPQTARNRAIDIIRESMALHINGDQHLASLCQYGVNEQRDSNWSFCTPAIGAGY

PRWWRPDEVGMPHQNRPQHGLAHTGEYLDGFGNKVYVYCTGNPEVATKRGRYERAHQKGS

GFGFVTIDTLKKTYTLDAYQFNADPTDGNPDNQFPGWPITLAQAENKGQNRDH

>H950DRAFT_00398

MGDDFSYADEFKTVDLAALQADIFELMTTSQDWWPADYGHYGPFFIRMAWHSAGTYRIAD

GRGGAASGTLRFAPLNSWPDNVNLDKARRLLWPIKQKYGKTLSWADLMVFTGNCALESMG

LKTFGFAGGREDVWEPEIDISWGAESEWLGDERYSGDRDLANPLGAVQMGLIYVNPEGPN

GTPDPLASAVDIRETFRRMAMNDEETVALIAGGHTFGKAHGAADPEQYVGPEPEGANLDD

QGLGWKNSFGTGNAGDTISSGLEGAWTTEPAKWDNNYFDNLFGYEWEQVKSPAGATQWTP

TDVSAQATVADAHDPDKKHAPMMFTTDMALKMDPVYGPISKRFHENPEEFADAFAKAWYK

LTHRDMGPVSRCLGPLVPEPQLWQDPIPETTHETISDEDVTELKTQILASDISLSQLVST

AWASATTFRATDNRGGANGARLRLAPQKDWDANVPAQLAGVLQVLEGIQSTFNSSQADGK

QVSLADLIVLGGTAAIEEAAKQAGTDVAVPFTAGRADASQEQTDVESFAVLEPAADGFRN

YAGADLGFPAEELLIDRAHLLTLTAPEMTVLVGGLRVLNVTAGDSGLGVFTKNPETLTND

FFVNLLDNSIKWQKSSKCEHFYEGCDRDSGEIKWMGTAVDLVFGSNSQLRAVAEAYACDD

SQQAFVVDFVAAWNKVMNLDRFDV

>H950DRAFT_00399

MFVYLLCFTSLVAQDKTGQNKATPVSHIKVVPGFEVELLYSVPSKDQGSWVNLCVDNKGR

IIVSDQFGGLYRMQPPAAGSPLDPASIETVPAPIRAVNGMVWAFDALYVGVNDYEQKISS

GLYRLTDSTGDDQLDKVELLREVKSRGDHGVHAVVPTPDGKSLFLITGNNSKPPIIEPTS

PVTQVWGEDHLLPSMPDGRGHNRGVLAPGGIIYKVSPDGKQFEAYASGFRNIFGGAINRD

GELFTYDADMEYDFNTPWYRPTRICLVTSGAEFGWRNGAGKRPVFYPDNLPGVLDIGPGS

PTGMTFGYGAKFPAKYQNALYSLDWSWGKLYAVHLKPDGAGYTATKEEFVTGAPLPITDA

MIHPGDGAMYFTIGGRRVQSGLYRVTYVGNESTRAVSTKPTVTAERQTRHMLEEFHGEPN

PKALNAAWPLLDHQDRLIRWAARMAVEHQPTEQWTERALQDPNPNTQVEALLALCRMTGI

CPQHRTDQSPPVDEAMGQKLIQALLAVNTAKLKPAQQLTLVRTLQITLIRFGRPEHAIVT

ELNQKFDPLFPADTFEENWLLCETLAWLESPTVAAKTLALIQNAATQEEQLQYARSLRML

QAGWNTELRTQYFNWFLKADSYRGGASFDIFLKFIRDDAVASLDAKSKTSLAQLLATKPV

RKSVIENLGAIFEGRETQDWSLDTLSAAANADLKDRNYDNGKRMFAAAACYACHRFNNQG

GITGPDLTSAGRRYSPHDLLDQIINPSKVINDQFSAVVVITDAGVIHTGVVVNLNGDSLM

LNTDLTNPNQQVRLNRNEIDEINISKVSPMPKGLLAKMTREEILDLVAYVISAGDSKHKV

FKE

>H950DRAFT_00400

DSTSVALIALNRNQNSRAIEVRDANELRKDAYPQILESLLNDTETDGSVGVLNAGILSYS

PLLEEQMLEHVVKHYRPHVVTLMLDCTDIGDDYHYSLEYDPQQLGGPFSGPDVTKPKPHF

GALWRFAKPLHPTLLAPFKLLDRLGGGYQPLDPFDYYKFRLLVAGKVETERFFIYRHPLD

VTRQYFDATWDTINRIAATCRQQDAEFILYIAPRYHHWNAKESPDNWETFAYGLEEPYQF

EIFNYFESKIDSAPFKIVNMLPTFQATQEYPLVFRTDPHWNPQGNRFVAHLLQRSLTNSQ

SK

**(B)**
